# Antimicrobial peptide-targeted photodynamic therapy for preventing periodontal plaque biofilm formation through the disruption of quorum sensing system

**DOI:** 10.1016/j.mtbio.2025.101970

**Published:** 2025-06-18

**Authors:** Wen Li, Fengqun You, Jie Yang, Deao Gu, Yuyang Li, Xuan Zhang, Leiying Miao, Weibin Sun

**Affiliations:** a Department of Endodontics, Nanjing Stomatological Hospital, Affiliated Hospital of Medical School, Research Institute of Stomatology, Nanjing University, Nanjing, China; b Shanghai Aiyou Biotechnology Center, Shanghai, China; c Department of Periodontics, Nanjing Stomatological Hospital, Affiliated Hospital of Medical School, Research Institute of Stomatology, Nanjing University, Nanjing, China; d Department of Orthodontics, Nanjing Stomatological Hospital, Affiliated Hospital of Medical School, Research Institute of Stomatology, Nanjing University, Nanjing, China; e Department of Prosthodontics, Nanjing Stomatological Hospital, Affiliated Hospital of Medical School, Research Institute of Stomatology, Nanjing University, Nanjing, China

**Keywords:** Metal–organic frameworks, Photodynamic therapy, Antimicrobial peptides, Inhibiting biofilms formation, LuxS/AI-2

## Abstract

Owing to high rates of antibiotic resistance, the elimination of periodontal plaque biofilms has become a significant clinical challenge. In this context, metal–organic framework (MOF)-based photodynamic therapy (PDT) has emerged as a novel antimicrobial treatment option. However, this therapeutic strategy suffers from drawbacks such as the insufficient generation of reactive oxygen species and the lack of targeted biofilm clearance, which greatly hinder its clinical application. Here, a multifunctional MOF-based nanocomposite (ICG@Uio-66-UBI) was developed by modifying MOFs (Uio-66-NH_2_) with an antimicrobial peptide (UBI29-41) to enhance PDT efficiency. Our findings showed that the UBI29-41 targets EPS and selectively binds to lipopolysaccharide (LPS) on bacterial surfaces via electrostatic interactions, enabling precise delivery of ICG-generated ROS under 808-nm near-infrared irradiation, which disrupts bacterial membranes and inhibits biofilm formation. Subsequently, UBI29-41 blocks LPS-TLR4 binding, suppressing NF-κB signaling and reducing pro-inflammatory cytokine production. Furthermore, the nanocomposite significantly downregulates the LuxS/AI-2 quorum sensing (QS) system, reducing AI-2 synthesis and virulence gene expression, thereby inhibiting biofilm formation. *In vivo* studies confirmed the platform's efficacy in inhibiting biofilm formation and preventing collagen degradation in gingival tissue. By synergistically combining targeted antimicrobial action, anti-inflammatory effects, and QS modulation, ICG@Uio-66-UBI represents a breakthrough in precision periodontal therapy, offering a potent solution for biofilm-associated infections.

## Highlights

• We first successfully developed multifunctional MOF-based nanocomposite (ICG@Uio-66-UBI) was developed by modifying MOFs (Uio-66-NH_2_) with an cationic antimicrobial peptide (UBI29-41) to enhance PDT efficiency.
• Our findings showed that the UBI29-41 could target both LPS and LTA on the surface of bacteria through electrostatic interactions. Meanwhile, the MOFs generated ROS under 808nm near-infrared irradiation, disrupting bacterial cell membranes and inhibiting biofilm formation.
• The potential mechanism by which ICG@Uio-66-UBI inhibits biofilm formation could involve the nanocomposite significantly downregulating the activity of the LuxS/AI-2 quorum sensing (QS) system and the expression of associated genes (*RadD, FadA, FomA, Fap2,RgpA, RgpB, HagA, HagB,* and *Kgp*).
• We showed that cationic antimicrobial peptide UBI29-41 specifically targets the LPS on the surface of bacteria and inhibiting the NF-κB signaling pathway, thereby ultimately exerting anti-inflammatory effects.
• This study offers a novel strategy for the precise treatment of periodontal disease and compensates for the issue of inadequate targeting in conventional periodontitis treatment. Since QS promotes biofilm formation and virulence expression, it can be considered a potential target against bacterial infection.

## Graphical abstract

A multifunctional MOF-based nanocomposite (ICG@Uio-66-UBI) was developed by modifying MOFs (Uio-66-NH_2_) with an antimicrobial peptide (UBI29-41) to remove periodontal plaque biofilms. UBI29-41 could target both lipopolysaccharide (LPS) and lipoteichoic acid (LTA) on the surface of bacteria through electrostatic interactions. Meanwhile, the MOFs generated ROS under 808-nm near-infrared irradiation, disrupting bacterial cell membranes and inhibiting biofilm formation. Subsequently, the interaction between UBI29-41 and LPS could inhibit NF-κB signaling pathway activation. Furthermore, the nanocomposite significantly down-regulated the activity of the LuxS/AI-2 quorum sensing system and the expression of related genes.

**Figure:**
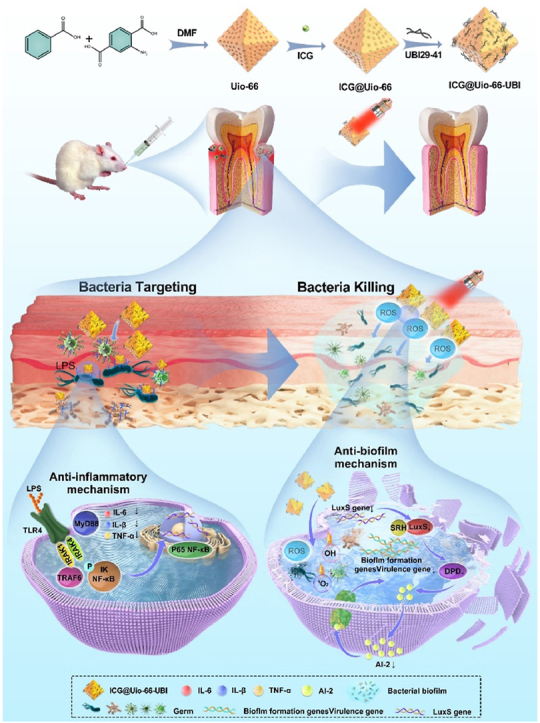
A multifunctional MOF-based nanocomposite (ICG@Uio-66-UBI) was developed by modifying MOFs (Uio-66-NH_2_) with an antimicrobial peptide (UBI29-41) to remove periodontal plaque biofilms. UBI29-41 could target both lipopolysaccharide (LPS) and lipoteichoic acid (LTA) on the surface of bacteria through electrostatic interactions. Meanwhile, the MOFs generated ROS under 808-nm near-infrared irradiation, disrupting bacterial cell membranes and inhibiting biofilm formation. Subsequently, the interaction between UBI29-41 and LPS could inhibit NF-κB signaling pathway activation. Furthermore, the nanocomposite significantly down-regulated the activity of the LuxS/AI-2 quorum sensing system and the expression of related genes.

## 1 Introduction

Periodontal infection is a chronic disease caused by bacteria and induces an inflammatory response in the host that results in periodontal tissue destruction and bone resorption [1,2]. Numerous studies have confirmed that in dental plaque—a complex microbial biofilm that contains a variety of gram-positive and gram-negative bacteria—the microorganisms and their metabolites interact with the host's immune defenses, contributing to an inflammatory response [3,4]. Thus, developing effective strategies to prevent plaque formation and inflammation is critical for periodontal disease management. The current treatment approach for periodontal disease typically involves mechanical debridement, including tooth brushing, or non-surgical periodontal treatment and antibiotic therapy to inhibit bacterial growth and reduce bacterial colonization [5]. However, complete plaque removal is challenging to achieve with traditional treatments owing to the presence of deep structures such as periodontal pockets and dental roots [6]. These limitations of traditional treatments have prompted the search for new alternatives to effectively control inflammation and inhibit the formation of plaque biofilms.

Antimicrobial photodynamic therapy (aPDT) is a relatively new and promising strategy for the treatment of several diseases, including peri-implantitis, periodontitis, and other oral lesions [7,8]. In aPDT, photosensitizers are excited by specific wavelengths of light and react with endogenous oxygen to produce cytotoxic reactive oxygen species (ROS), which damage bacterial plasma membranes and DNA and induce cell death [9]. However, the clinical application of free photosensitizers is limited by poor water solubility, low photostability, and inefficient ROS generation [10]. To address these challenges, nanotechnology-based aPDT strategies have gained attention due to the superior water solubility of nanoparticles (NPs) and their ability to regulate ROS release. Metal–organic frameworks (MOFs), particularly Uio-66, have shown promise as photosensitizer carriers because of their tunable pore sizes, large surface areas, and excellent biocompatibility [[11], [12], [13]]. Uio-66-NH_2_, an amino-modified variant, enhances ROS production even under low oxygen conditions, making it effective against periodontal pathogens resistant to conventional PDT [14]. Nevertheless, exogenous MOF-based nanomaterials lack mechanisms for actively targeting bacteria and thereby trigger an immune response. Thus, it is particularly important to develop nano-delivery systems that target both periodontal pathogens and inflammation.

Cationic antimicrobial peptides (CAMPs) are key components of the innate immune system and represent a potential source of novel antibiotics. In addition to their direct antimicrobial effects, CAMPs modulate immune responses, making them attractive for therapeutic applications [15]. Recent studies have explored the use of CAMPs to achieve targeted antimicrobial effects through interactions with negatively charged bacterial components, such as lipopolysaccharide (LPS) in gram-negative bacteria and lipoteichoic acid (LTA) in Gram-positive bacteria [16,17]. These interactions disrupt bacterial membranes and enhance the penetration of antimicrobial agents [[18], [19], [20], [21]]. Notably, LPS activates Toll-like receptor4 (TLR4), triggering MyD88-dependent NF-κB signaling. This pathway upregulates inflammatory mediators, including interleukin-6 (IL-6), tumor necrosis factor-α (TNF-α), and cyclooxygenase-2 (COX-2) [[22], [23], [24]]. CAMPs such as UBI29-41 not only neutralize LPS but also inhibit NF-κB activation, offering dual therapeutic benefits [25,26]. With exceptional thermal stability and pH resilience, UBI29-41 is uniquely suited for constructing multifunctional nanoparticles [27].

In recent years, with the development of nanotechnology, treatment strategies such as chemodynamic therapy, phototherapy, and sonodynamic therapy (SDT) have gained prominence as alternatives to antibiotic therapy [[28], [29], [30]]. However, the double-membrane structure of Gram-negative bacteria, including *Fusobacterium nucleatum* and *Porphyromonas gingivalis*, limits the penetration of ROS, reducing the effectiveness of single therapies [31,32]. Combining CAMPs with aPDT presents a synergistic strategy that targets both bacterial membranes and metabolic pathways, thereby improving treatment efficiency.

In this study, we developed a dual-functional nano-platform combining aPDT with the targeting peptide UBI29-41 to achieve enhanced antimicrobial and anti-inflammatory effects under 808nm near-infrared (NIR) irradiation. Unlike previous studies that primarily focused on biofilm inhibition, our work investigates the underlying mechanisms of action. Bacterial biofilm formation and virulence are regulated by quorum sensing (QS), a communication system mediated by signaling molecules such as autoinducer-2 (AI-2), synthesized by the LuxS enzyme [33,34]. We hypothesize that our nanoplatform could disrupt the LuxS/AI-2 QS system, thereby inhibiting both biofilm formation and virulence factor expression.

**Fig. 1:**
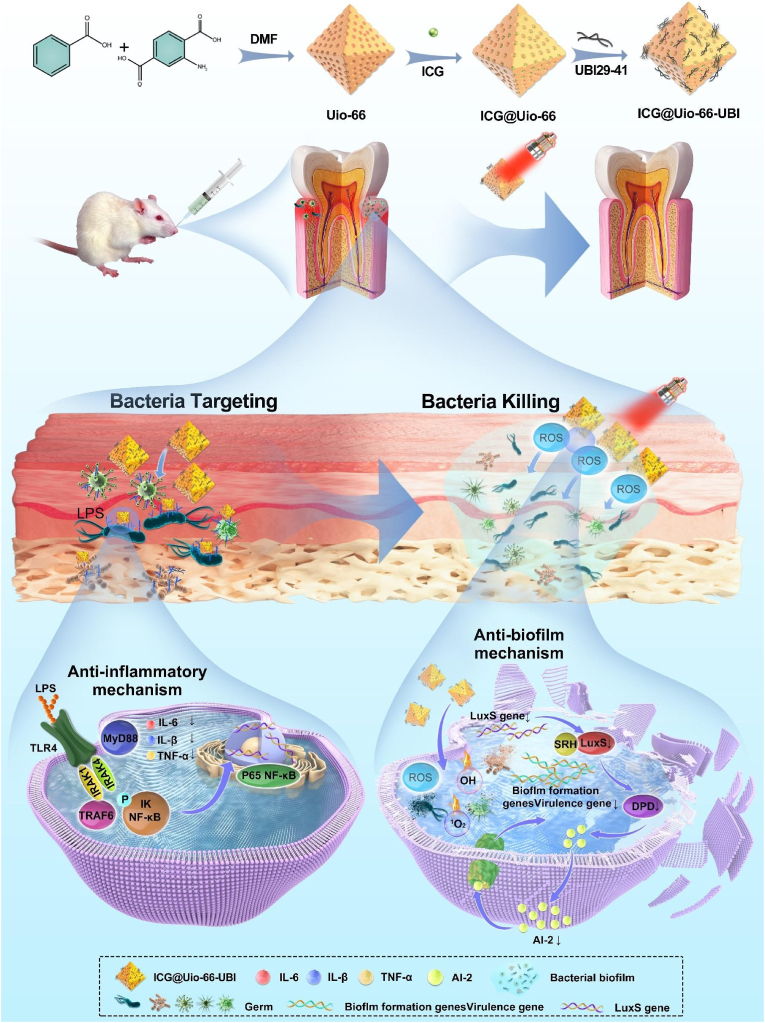
Schematic illustration of synthesis of ICG@Uio-66-UBI NPs and their remarkable ability to enhance target anti-biofilm and immunomodulation against periodontal diseases.

To test this hypothesis, we encapsulated the photosensitizer indocyanine green (ICG) within Uio-66 to form a core-shell structure and modified the surface with UBI29-41, creating a NIR therapeutic platform (Fig. 1). This platform, termed ICG@Uio-66-UBI, combines targeted aPDT with anti-inflammatory effects, demonstrating favorable biosafety both *in vitro* and *in vivo*. Our design achieves the following objectives: 1) Specific targeting of pathogenic bacteria through interactions between UBI29-41 and negatively charged LPS/LTA, while ROS generated by ICG under NIR irradiation penetrates bacterial membranes, enhancing aPDT efficacy. 2) Suppression of the NF-κB pathway by UBI29-41, which inhibits LPS-induced inflammation and modulates the local inflammatory microenvironment. 3) Regulation of the LuxS/AI-2 QS system to reduce virulence factor secretion and inhibit biofilm formation. This platform, termed ICG@Uio-66-UBI, combines targeted aPDT with anti-inflammatory effects, demonstrating favorable biosafety and synergistic efficacy *in vitro* *and in vivo.* This work advances periodontal therapy by combining bacterial targeting, inflammation control, and QS inhibition—a multifunctional platform addressing the complex nature of periodontal disease. By overcoming the limitations of conventional treatments, our platform offers a precise, mechanism-driven approach for managing deep-seated dental infections.

## 2 Materials and methods

### 2.1 Preparation of ICG@Uio-66 composites

To synthesize the ICG@Uio-66 composites, 90 mg of amino terephthalic acid, 115 mg of zirconium chloride, 1.8 g of benzoic acid, and 80 μL of concentrated hydrochloric acid were dissolved in 35 mL of N,N-dimethylformamide (DMF) under continuous stirring. The homogeneous mixture was transferred to a hydrothermal kettle and heated at 120 °C for 10 h. After cooling to room temperature, the mixture was centrifuged at 15,000 ×g for 30 min, and the resulting precipitate was washed three times with ethanol to remove impurities. To ensure complete removal of unreacted organic solvents, the material was soaked in ethanol for 3 days. The purified product was then centrifuged, and the collected precipitate was freeze-dried for subsequent use. For the loading of ICG, ICG (100 mg) was mixed with Uio-66 (50 mg) in ethanol (200 mL) and stirred for 24 h. All subsequent experiments were performed using ICG-loaded nanoplatforms as intended.

### 2.2 Introduction of UBI target

The carboxyl groups on the UBI peptide were activated by dissolving UBI (1 mg/mL) in deionized water and adding 1-ethyl-3-(3-dimethylaminopropyl) carbodiimide (EDC) (5 mg/mL, pH 5.6). The solution was stirred at 37 °C for 2 h to facilitate activation. Next, an equal volume of moxifloxacin-adsorbed Uio-66 (2 mg/mL) was added to the activated UBI solution. To promote covalent bonding between the amino groups of Uio-66 and the carboxyl groups of UBI, 5 mg/mL of N-hydroxysuccinimide (NHS) solution was introduced, and the mixture was gently agitated at room temperature for 24 h. After the reaction, the resulting material was collected, lyophilized, and stored at -80 °C for further use.

### 2.3 Characterization of NPs

The ultrastructure of drug-free Uio-66, ICG@Uio-66, and ICG@Uio-66-UBI samples was examined using field emission transmission electron microscopy (TEM). NP size and zeta potential were measured using the Zetasizer Nano instrument (Malvern Instruments, Malvern, UK). The solutions (25 mg/mL) were then transferred to 1 mL polystyrene cuvettes for analysis. Nitrogen adsorption–desorption was conducted using a JW-BK112 surface area and porosity analyzer (JWGB Sci & Tech Co., Beijing, China). The surface area was determined by employing the Brunauer–Emmett–Teller (BET) model, while the cumulative pore volume was calculated from the adsorption branch of the isotherm by employing the Barrette–Joyner–Halenda (BJH) model. Thermogravimetric analysis (TGA) was conducted in ambient air using an STA 6000 simultaneous thermal analyzer (PerkinElmer, Waltham, MA, USA) over a temperature range of 30–800 °C at a heating rate of 5 °C/min. UV–vis absorption spectra were obtained using a Lambda 950 UV–vis spectrophotometer (PerkinElmer, Lambda 950).

### 2.4 Photothermal performance evaluation of ICG@Uio-66-UBI NPs

The photothermal performance of ICG@Uio-66-UBI NPs was evaluated in vitro by measuring temperature changes under 808 nm laser irradiation. NPs were prepared at concentrations of 2.5, 5, 10, 20, and 30 μg/mL and exposed to laser power densities of 0.5, 1.0, and 2.0 W/cm^2^. Samples were placed in quartz dishes and irradiated for 10 min, with FLIR thermal imaging used to monitor temperature changes. Real-time temperature measurements were recorded every 2 min. Each experiment was performed in triplicate to ensure reproducibility. Additionally, the photothermal performance of ICG@Uio-66-UBI NPs at a fixed concentration was compared across different power densities (0.5, 1.0, and 2.0 W/cm^2^) using the same experimental protocol.

### 2.5 Detection of singlet oxygen (^*1*^*O*_*2*_) from ICG@Uio-UBI nanocomposites

The singlet oxygen (^*1*^*O*_*2*_) generation capability of ICG@Uio-66 and ICG@Uio-66-UBI was assessed using 3,3′,5,5′-tetramethylbenzidine (TMB) as a substrate. Enzyme markers, such as horseradish peroxidase (HRP), were added to NP solutions (2.5, 5, 10, 20, and 30 μg/mL) to facilitate binding with target molecules (e.g., antigens or antibodies). TMB solution was then added, and 1 mL of the mixture was irradiated with 808 nm near-infrared light at 1 W/cm^2^ for 10 min. The absorbance of the resulting blue product was measured at 620–650 nm using UV–Vis spectrophotometry to quantify reaction intensity and target molecule concentration.

To further evaluate ^*1*^*O*_*2*_ generation, methylene blue (MB) was used as a substrate. A 1 mL aqueous solution containing MB (1 mM) and ICG@Uio-66-UBI (30 μg/mL) was irradiated with 808 nm NIR light at 1 W/cm^2^ for 0, 5, 10, 15, 20, and 30 min. UV–Vis absorption spectra were recorded at 664 nm to monitor MB degradation, which correlates with ^1^O_2_ production.

### 2.6 Cytotoxicity assay

L929 fibroblasts and human gingival fibroblasts (HGFs)were used to assess the cytocompatibility of Uio-66, ICG@Uio-66, and ICG@Uio-66-UBI NPs as aPDT agents for periodontal disease treatment. Cells were cultured in DMEM supplemented with 10% fetal bovine serum (FBS) and 1% penicillin–streptomycin (100 g/mL streptomycin and 100 U/mL penicillin). Then, the cells were incubated in a constant temperature incubator at 37 °C in 5 % CO_2_. Finally, the L929 cells were seeded at a density of 2.5 × 10^3^ cells per well in 96-well plates for in vitro culture. The cells were incubated with various concentrations of Uio-66, ICG@Uio-66, and ICG@Uio-66-UBI (0 μg/mL to 50 μg/mL) for 24 h and 72 h. The viability of the cells at 24 h and 72 h was determined using a Cell Counting Kit-8 (CCK-8) assay and the optical density (OD) was measured using the SpectraMax M3 (Molecular Devices, USA) microplate reader at 450 nm.

### 2.7 Bacterial culture and biofilm formation

For this study, *Porphyromonas gingivalis* (*P. gingivalis,* ATCC 33277), *Fusobacterium nucleatum* (*F. nucleatum,* ATCC 10953), *and Streptococcus gordonii* (*S. gordonii,* ATCC10558) were procured from the American Type Culture Collection (ATCC, Manassas, VA) for single-species biofilm formation*.* After culturing *P. gingivalis, F. nucleatum*, and *S. gordonii* in anaerobic conditions (5% CO_2_, 10% H_2_, and 85% N_2_) at 37 °C for 3–7 days, the microbial concentration was adjusted to 10^8^ colony-forming units (CFU)/mL. Both types of bacteria were grown on Brain Heart Infusion (BHI) medium at 37 °C*.* The AI-2 reporter strain *V. harveyi BB170* (ATCC BAA-1117) was aerobically cultured in 2216e medium at 30 °C with shaking.

### 2.8 Construction of saliva membrane

Saliva samples were collected from volunteers without active caries and no history of antibiotic use within the past three months. The study protocol was approved by the Ethics Committee of Nanjing Stomatological Hospital (Approval No. NJSH-202023NL-43). The samples were filtered using bacterial filters, and hydroxyapatite slices were immersed in the filtered saliva for 2 h to form a salivary film. After incubation, the supernatant was discarded.

### 2.9 In vitro assessment of the efficacy of NPs against single-species biofilms

For the in vitro assessment of NPs efficacy against single-species biofilms, seven groups were set up as follows: control, control + NIR, Uio-66, ICG@Uio-66, ICG@Uio-66-UBI, ICG@Uio-66 + NIR, and ICG@Uio-66-UBI + NIR. Then, we placed hydroxyapatite slices that had been coated with a saliva film were placed at the bottom of a 24-well plate. *P. gingivalis*, *F. nucleatum* and *S.* *gordonii* were inoculated onto separate hydroxyapatite slices (10^8^ CFU/mL) and were treated with various NPs (30 μg/mL), followed by NIR exposure (1 W/cm^2^, 10 min) for 3 days. The control group received neither NP treatment nor NIR exposure. To evaluate the efficacy of different NPs in inhibiting biofilm formation, live/dead fluorescent staining and standard plate counting assays were carried out. Moreover, biofilm biomass and extracellular polysaccharide production were quantified. Detailed procedures are listed in the Supporting Information.

### 2.10 Detection of bacterial cell membrane permeability

To evaluate the permeability of bacterial cell membranes following treatment with different nanoparticles (NPs), the release of nucleic acids from the cytoplasm was measured. Three bacterial species—*P*. *gingivalis, F*. *nucleatum,* and *S*. *gordonii*—were cultured to the logarithmic phase and adjusted to a concentration of 1 × 10^8^ CFU/mL. Equal volumes of the experimental materials, namely Uio-66, ICG@Uio-66, and ICG@Uio-66-UBI (each at a concentration of 60μg/mL), were added at twice the concentration of the bacterial suspension. The bacterial suspensions were then irradiated with an 808 nm NIR laser. The bacterial suspensions were immediately collected and filtered through a 0.22 μm filter to remove bacterial cells. The supernatant was subsequently diluted, and the optical density (OD) at 260 nm was measured using a UV–Vis spectrophotometer.

### 2.11 In vitro assessment of the efficacy of NPs against multi-species biofilms

Seven identical groups of multi-species biofilms were established using the same procedure used for single-species biofilm experiments. Bacterial suspensions of *F. nucleatum* (10^8^ CFU/mL), *P. gingivalis* (10^8^ CFU/mL), and *S. gordonii* (10^8^ CFU/mL) were combined in equal volumes to create a mixed bacterial suspension, which was then placed onto hydroxyapatite slices in 24-well plates. The suspensions were treated with various NPs (30 μg/mL) and exposed to NIR irradiation (808 nm, 1 W/cm^2^, 10 min) for 3 days. Live/dead fluorescent staining and scanning electron microscopy (SEM) were performed to examine the impact of NPs on multi-species biofilms.

### 2.12 Mechanism underlying the antibacterial effects of ICG@Uio-66-UBI

Log-phase *F. nucleatum* cells were diluted to a concentration of 1 × 10^8^ CFU/mL and treated with ICG@Uio-66-UBI NPs (30 μg/mL), followed by NIR exposure (808 nm, 1 W/cm^2^, 10 min), for 3 days. The control group did not receive any NP or NIR treatment. The bacterial suspensions were collected for total RNA extraction. Sequencing was performed on the Illumina NovaSeq 6000 platform by Suzhou Panomik Biomedical Technology Co. Ltd. The concentration and purity of the extracted RNA were evaluated using an Agilent 2100 Bioanalyzer. After rRNA removal, RNA fragmentation, cDNA synthesis, and polymerase chain reaction (PCR) enrichment, high-quality clean data were obtained for subsequent analyses. The reference genome was established using Bowtie2, and the filtered reads were aligned to the reference genome. The gene read count values were determined using HTSeq (version 0.6.1p2) and represented the original expression levels of the respective genes. The gene expression levels in different samples were normalized using FPKM (fragments per kilobase of exon per million fragments mapped) values. DESeq (version 1.20.0) was employed to identify differentially expressed genes (DEGs), and genes with |log2FoldChange| > 1 and a *p* value of <0.05 were considered significant. GO enrichment analysis was performed using topGO to determine the main biological functions of the DEGs, and Gene Ontology (GO) terms with a *p* value of less than 0.05 were considered significantly enriched. Kyoto Encyclopedia of Genes and Genomes (KEGG) pathway enrichment analysis was performed using clusterProfiler (version 3.4.4) software to identify significantly enriched pathways (*p* < 0.05).

### 2.13 mRNA expression of LuxS

LuxS is a critical enzyme involved in regulating bacterial biofilm formation. As the bacterial density increases, LuxS induces the secretion of the AI-2 signal molecule, which in turn promotes intercellular signaling and bacterial aggregation, leading to the formation of biofilms. Therefore, the expression of *LuxS* gene — was quantitatively determined using the qRT‒PCR technique. Primers for the target genes were custom synthesized by GenScript (Nanjing, China); specific information is provided in Table S2. The expression of mRNA was assessed using the 2^−ΔΔCt^ method. The 16S rRNA gene was utilized as the internal reference. All experiments were repeated three times.

### 2.14 AI-2 inhibitory activity of ICG@Uio-66-UBI

The concentration of *V. harveyi* in fresh 2216e medium BB170 was adjusted to 1 × 10^6^ CFU/mL. The *F. nucleatum* suspension (1 × 10^8^ CFU/mL) was subsequently combined with ICG@Uio-66-UBI or left untreated, and the samples were divided into three groups: (1) Control group: The *F. nucleatum* suspension was cultured in a ICG@Uio-66-UBI-free medium; (2) ICG@Uio-66-UBI group: The *F. nucleatum* suspension and 30 μg/mL ICG@Uio-66-UBI were incorporated into the medium; (3) ICG@Uio-66-UBI + NIR group: The *F. nucleatum* suspension and 30 μg/mL ICG@Uio-66-UBI NPs were incorporated into the medium and exposed to NIR irradiation (808 nm, 1 W/cm^2^, 10 min). Subsequently, all samples were incubated at 37 °C for 1–12 h. Meanwhile, 1 mL of the culture medium was harvested at 4, 6, 8, 10, and 12 h and subsequently centrifuged for 10 min to obtain the supernatant. To assess the AI-2 activity in each experimental group, 90 μL of BB170 was incubated with 10 μL of the AI-2 supernatant (prepared using the aforementioned experimental samples) for 6 h at 28 °C. The bioluminescence values were measured at 490 nm using a Promega Luminometer. This experiment was conducted independently three times.

### 2.15 Expression of virulence factors in *F. nucleatum*

*F. nucleatum* is an important bridge bacterium in the early and late stages of plaque pathogen colonization, playing an important role in biofilm formation. Therefore, the expression of its virulence factors — the *Fap2*, *FomA*, *RadD*, and *FadA* genes — was quantitatively determined using the RT‒qPCR technique. Primers for the target genes were custom synthesized by GenScript (Nanjing, China); specific information is provided in Table S2. The expression of mRNA was assessed using the 2^−ΔΔCt^ method. The 16S rRNA gene was utilized as the internal reference. All experiments were repeated three times.

### 2.16 Extraction of inflammatory stimulants and experimental grouping

The anti-inflammatory effects of ICG@Uio-66-UBI were assessed using an inflammatory model based on RAW 264.7 murine macrophages. To prepare the inflammatory stimulus, a *P*. *gingivalis* suspension was adjusted to an optical density (OD600) of 0.2 and centrifuged at 7000 rpm for 10 min to collect bacterial pellets. The pellets were washed with phosphate-buffered saline (PBS) to remove residual culture medium, resuspended in deionized water, and mixed with an equal volume of 90 % phenol solution. The mixture was heated at 65 °C for 15–30 min, followed by centrifugation at 7000 rpm for 20 min at 4 °C to isolate lipopolysaccharide (LPS). The aqueous phase was dialyzed to remove residual phenol and lyophilized to obtain LPS powder, with its concentration quantified using ultraviolet spectrophotometry at 260 nm.

RAW 264.7 cells were seeded at a density of 1.2 × 10^5^ cells per well in a 6-well plate and incubated in 2 mL of medium for 24 h. Subsequently, 200 μL of the 10 μg/mL LPS was added to each well, except for the control group, which received normal medium. After 6 h, ICG@Uio-66 or ICG@Uio-66-UBI NPs were added to the medium at a concentration of 30 μg/mL, while the control group remained untreated. The experimental groups were categorized as follows: 1) Control group: Cells cultured in LPS-free medium. 2) ICG@Uio-66 group: Medium supplemented with 10 μg/mL LPS and 30 μg/mL ICG@Uio-66. 3) ICG@Uio-66-UBI group: Medium supplemented with 10 μg/mL LPS and 30 μg/mL ICG@Uio-66-UBI. 4) ICG@Uio-66 + NIR group: Medium supplemented with 10 μg/mL LPS and 30 μg/mL ICG@Uio-66, with NIR irradiation (808 nm, 1 W/cm^2^, 10 min) applied twice daily. 5) ICG@Uio-66-UBI + NIR group: Medium supplemented with 10 μg/mL LPS and 30 μg/mL ICG@Uio-66-UBI, with NIR irradiation (808 nm, 1 W/cm^2^, 10 min) applied twice daily.

Following treatment, all groups were cultured for an additional 12 h to evaluate the anti-inflammatory effects.

### 2.17 Gene and protein expression of inflammatory factors in RAW 264.7 cells

The expression of proinflammatory cytokine genes *IL-6*, *TNF-α*, *IL-1β*, *IL-8*, *iNOS*, and *COX-2* were investigated via qRT-PCR. The qPCR primers were designed as shown in Table S3. Relative gene expression was assessed using the 2^−ΔΔCt^ method.

Immunofluorescence staining for proinflammatory cytokines (IL-6, TNF-α, and iNOS) was performed as follows: RAW 264.7 cells were fixed with 4 % paraformaldehyde (PFA) for 20–30 min and incubated in 5 % blocking buffer (BSA). Then, the cells were incubated with mouse anti-IL-6, anti-TNF-α, and anti-iNOS primary antibodies (1:400) overnight at 4 °C. Immunofluorescence images were collected using confocal laser scanning microscopy (CLSM) after the cells were incubated with the secondary antibody (1:400) and DAPI. All experiments were repeated three times.

### 2.18 Evaluation of NF-κB activation in macrophages by NPs

The transposition of NF-κB/p65 in RAW 264.7 cells was tested through immunofluorescence staining. Immunofluorescence staining for NF-κB/p65 was performed as described for proinflammatory cytokines in section 2.11. The cells were fixed with 4% PFA for 20–30 min, permeabilized with 0.1% TritonX-100 for 5 min, and blocked with 5% BSA for 30 min. Subsequently, the cells were incubated overnight at 4 °C with mouse anti-NF-κB/p65 primary antibodies (1:400), followed by incubation with the secondary antibodies (1:800) and DAPI. The images were randomly acquired using CLSM.

### 2.19 Regulating the NF-κB pathway

RAW 264.7 cells (1 × 10^6^/well) were pretreated with 30 μg/mL ICG@Uio-66-UBI and 10 μg/mL LPS for 0,15,30,60,90 min. The protein level of p65, IκBα and TLR4 was quantitively investigated via western blot. In brief, the total protein was obtained in RIPA lysis buffer with PMSF with 30 min. Following a series of standardized procedures, including electrophoresis, membrane transfer, blocking, and incubation with primary antibodies (anti-p-p65, anti-p-IκBα, anti-TLR4) and HRP-conjugated secondary antibody incubation, the proteins were visualized with an ECL kit by a gel imaging analysis system. β-actin and α-tubulinwas used as internal control.

### 2.20 Development of a periodontal inflammation model and therapeutic interventions

Male Wistar rats (∼180 g) aged 7–8 weeks were utilized to establish a model of periodontal inflammation *in vivo.* All animal experiments were explicitly approved by the Nanjing Stomatological Hospital Ethics Committee (#IACUC-D2304013). The Wistar rats were anesthetized with 10 % chloral hydrate (4 mL/kg of body weight). Then, a 100 μL suspension of *P. gingivalis* (OD_600_ = 0.2) was injected into the vestibular groove of the mandibular incisor for 14 consecutive days. After the model was established, rats were randomized into five groups: Inflammatory control, ICG@Uio-66, ICG@Uio-66-UBI, ICG@Uio-66 + NIR, and ICG@Uio-66-UBI + NIR. Then, based on group allocation, various NPs (30 μg/mL, 0.1 mL) were injected at the same site as the *P. gingivalis* injection, and 808 nm NIR irradiation ( 1 W/cm^2^, 10 min) was provided for 3 days. The control group received an equivalent dosage of PBS instead.

### 2.21 In vivo evaluation of antibacterial effects

At the end of the treatment period, the bacterial counts of treated tissue were assessed using a standard plate counting assay to evaluate the antibacterial efficacy of NPs against periodontal inflammation. In brief, swabs were gently inserted into the infected site for 10 s and immediately placed in 2 mL of BHI medium and incubated for 48 h. The bacterial suspensions were diluted using the gradient dilution method and cultured on blood agar plates before the enumeration of bacterial colonies.

### 2.22 In vivo fluorescence imaging

For the *in vivo* evaluation of the ROS levels in different groups, fluorescence imaging of live animals was performed following the administration of NPs. The ROS probe DCFH-DA (1.8 mg/kg) was initially administered via local injection at the treatment site. This was followed by a 30-min incubation period. Subsequently, an imaging system for small animals (excitation filter: 465 nm; emission filter: 520 nm; ColdSpring) was used to acquire images.

### 2.23 Histological evaluation

All rats were euthanized, and the gingival tissues at the treatment sites were excised after 3 days of treatment. The specimens were fixed in 4% PFA for histological analysis. The inflammatory infiltration of the gingival tissue was further evaluated using hematoxylin and eosin (H&E) staining, Masson's trichrome staining, and immunohistochemistry (IHC). A light microscope (Olympus) was used to view the H&E-stained slides. At 100× magnification, the inflamed regions were identified, and collagen degradation was evaluated. IHC staining was employed for the evaluation of proinflammatory cytokine levels in the tissue following treatment with various NPs. Rabbit anti-IL-6 (GB11117, Servicebio) and rabbit anti-TNF-α (GB11188, Servicebio) were used as the primary antibodies (1:400). Stained regions were observed at 100× and 400× magnification and the average fluorescence intensity was measured using Image J.

### 2.24 Statistical analysis

GraphPad Prism 8.6, Origin 2018, and Image J-Fiji software were used for statistical analyses. One-way analysis of variance (ANOVA) was carried out to determine the significance of differences among groups. A value of p < 0.05 was deemed to be statistically significant.

## 3 Results and discussion

### 3.1 Preparation and characterization of ICG@Uio-66-UBI NPs

To construct the multifunctional nanoplatform, a stepwise strategy was employed integrating photodynamic functionality, bacterial targeting, and structural support. First, Uio-66-NH_2_ was synthesized as a zirconium-based MOF with high porosity and abundant surface amine groups. ICG, a negatively charged NIR-absorbing photosensitizer, was subsequently loaded into the MOF via electrostatic interactions and physical encapsulation, endowing the system with PDT capability. Finally, the antimicrobial peptide UBI29-41, known for its strong affinity to bacterial membranes due to its cationic and amphipathic properties, was covalently conjugated to the MOF surface through EDC/NHS-mediated amide bond formation with the terminal amine groups. This sequential modification results in a well-defined nanocomposite—ICG@Uio-66-UBI—that simultaneously possesses (i) stable MOF architecture for structural integrity and drug loading, (ii) ICG-mediated ROS generation upon NIR irradiation, and (iii) UBI29-41-mediated bacterial targeting.

**Fig. 2:**
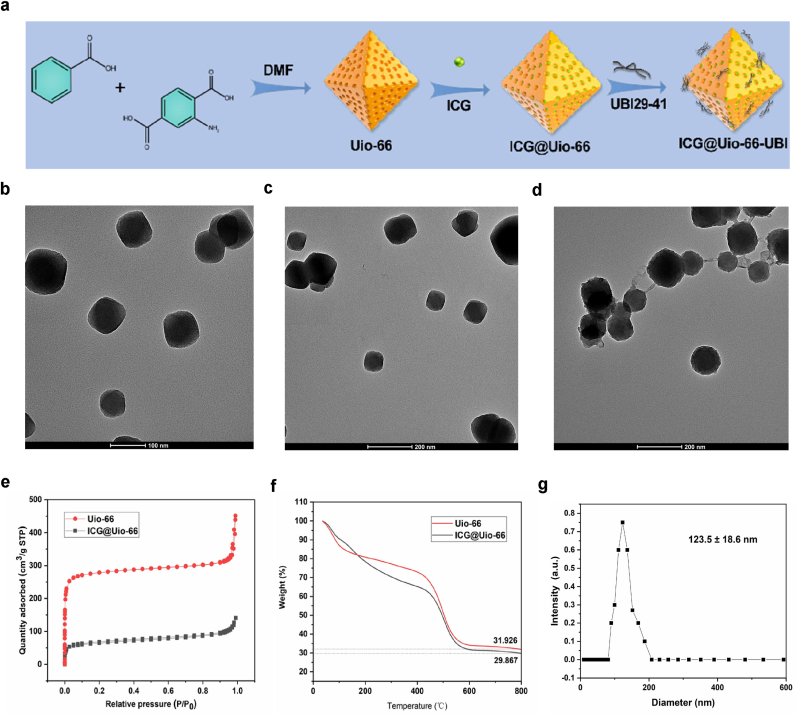
Characterization of Uio-66, ICG@Uio-66 and ICG@Uio-66-UBI NPs. (a) Schematic illustration of the synthesis of ICG@Uio-66-UBI NPs. (b–d) TEM images of various NPs, including Uio-66, ICG@Uio-66 and ICG@Uio-66-UBI NPs. (e) Nitrogen adsorption-desorption isotherms of Uio-66, ICG@Uio-66 NPs. (f) TGA of ICG@Uio-66 and ICG@Uio-66 NPs. (g) The size distribution of ICG@Uio-66-UBI NPs.

**Fig. 3:**
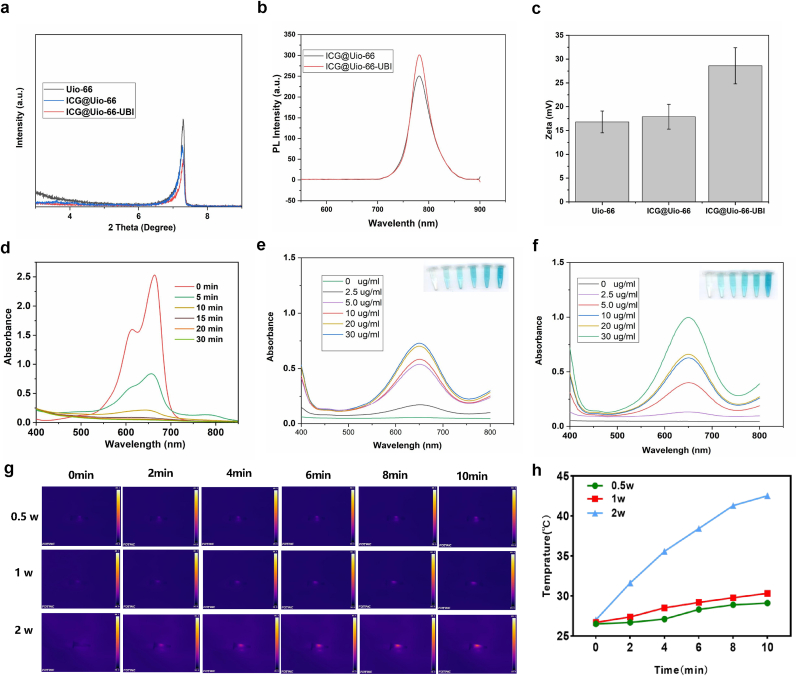
Characterization and in vitro evaluation on the ROS generation of NPs. (a) XRD pattern of various NPs. (b) UV–Vis spectra of ICG@Uio-66 and ICG@Uio-66-UBI NPs. (c) Zeta potential of different NPs. (d) UV–Vis absorption spectra of ICG@Uio-66-UBI mixed MB at different time. (e) UV–vis spectra of different concentrations of ICG@Uio-66 NPs. (f) UV–Vis spectra of different concentrations of ICG@Uio-66-UBI NPs. (g) Infrared thermal images of ICG@Uio-66-UBI at different light intensity under an 808 nm laser (0.5 W/cm^2^, 1 W/cm^2^, 2 W/cm^2^, 10 min). (h) Temperature of ICG@Uio-66-UBI at different light intensity under an 808 nm laser (0.5 W/ cm^2^, 1 W/cm^2^, 2 W/cm^2^, 10 min).

High-resolution TEM images (Fig. 2b–d) revealed that the particles of the synthesized Uio-66 material were uniformly dispersed, and the dispersibility of the material remained good even after loading ICG. Furthermore, after modifying the surface of the material with UBI, a significant rough layer appeared. The NP sizes were further examined by dynamic laser scattering (DLS). Similar to TEM observations, DLS demonstrated the size of the hydrated particles was primarily distributed around 123.5 ± 18.6 nm (Fig. 2g), this specific size enables effective interaction with bacterial cells, thereby enhancing its antimicrobial efficacy. X-ray diffraction (XRD) (Fig. 3a) and the BET model were employed to analyze specific surface area and further characterize the structure of Uio-66 before and after ICG loading. The small-angle XRD data indicated that pure Uio-66, ICG@Uio-66, and ICG@Uio-66-UBI all exhibited a diffraction peak at 7.3°. The nitrogen adsorption–desorption isotherms of Uio-66 and ICG@Uio-66 demonstrated type-I isotherm behavior, indicating the presence of micropores (Fig. 2e). Nevertheless, the BET surface area and pore volume of ICG@Uio-66 (1523.43 m^2^ g^−1^ and 0.46 cm^3^ g^−1^, respectively) were lower than those of pure Uio-66 (1756.65 m^2^ g^−1^ and 0.59 cm^3^ g^−1^, respectively), we hypothesize that the predominant factor is the filling effect induced by ICG, which may lead to the compression or deformation of certain pores. Alternatively, the incorporation of ICG might disrupt the crystallization process of Uio-66, thereby diminishing the material's internal structural order and yielding a more irregular pore architecture. Despite the observed reductions in specific surface area and pore volume, this composite framework could potentially offer distinctive performance benefits in realms like photocatalytic degradation. Fig. 3c shows that shows that the nanocomposite exhibits a Zeta potential of +13.6 mV indicating that while the incorporation of negatively charged ICG molecules does lead to a partial reduction in surface charge compared to bare Uio-66-NH_2_ (typically ∼16.8 mV), the composite remains positively charged overall. Importantly, the observed increase in Zeta potential after subsequent conjugation with UBI29-41 is attributed to the positively charged nature of the peptide itself, which contains multiple basic residues such as lysine and arginine. This modification offsets the slight charge reduction from ICG and results in a final surface potential higher than that of the ICG-loaded MOF alone.Since periodontal pathogens carry a negative charge on their surfaces, this provides a favorable condition for the binding of this nanoplatform to periodontal pathogens. TGA showed that the loading rate of ICG was about 2 wt% (Fig. 2f).

To confirm the photocatalytic activity of ICG@Uio-66 and ICG@Uio-66-UBI, standard assays based on the oxidation of TMB were performed. Since the presence of ROS facilitates the catalytic conversion of TMB, the reaction leads to the formation of a blue hue in an acetate buffer solution. As shown in (Fig. 3e and f and S1b), the rapid oxidation of TMB was observed in the presence of ICG@Uio-66 and ICG@Uio-66-UBI NPs. With increasing concentrations of ICG@Uio-66 and ICG@Uio-66-UBI NPs, a progressive deepening in the coloration of the post-reaction solution is observed, which serves as a visual indicator of the escalating concentration of ROS produced. However, the color of MB faded upon reaction with ROS (Fig. 3d). These findings conclusively indicate that the material exhibits significant photocatalytic activity. The photothermal effect of ICG@Uio-66-UBI is shown in Fig. 3g and h. Notably, the solution temperatures rose from 26.5 °C to 29.1 °C under 0.5 W/cm^2^ and from 26.7 °C to 30.3 °C under 1 W/cm^2^ of near-infrared irradiation. The resultant temperature increments were found to be modest. This suggests that ICG@Uio-66-UBI possesses thermal stability, and its mechanism of action is attributed to the photodynamic generation of ROS, rather than photothermal effects. Thus, NIR with a power density of 1 W/cm^2^ is suitable for conducting the follow-up experiments.

### 3.2 Biosafety assessment of different NPs

To assess the clinical feasibility of Uio-66, ICG@Uio-66, and ICG@Uio-66-UBI (Fig. S3), the in vitro biosafety of these nanoparticles (NPs) was evaluated using the CCK-8 assay in a mouse fibroblast cell line (L929). The results indicated that cell viability remained above 90% after 24 h when the NPs concentration was ≤40 μg/mL. However, a significant reduction in cell viability was observed at concentrations exceeding 40 μg/mL. After 72 h, cell viability was markedly inhibited at 40 μg/mL for all NPs (Fig. S3a–c). Similar trends were observed in HGFs using the CCK-8 assay (Fig. S3d–f). Based on these findings, 30 μg/mL was identified as a biosafe concentration for subsequent experiments. *In vivo* toxicity was further evaluated through histological examination of major organs, including the heart, liver, spleen, lungs, and kidneys. No significant histological changes were observed following treatment with the NPs (Fig. S3g), confirming their biocompatibility at low concentrations. These results demonstrate that Uio-66, ICG@Uio-66, and ICG@Uio-66-UBI exhibit excellent biocompatibility and hold significant promise for clinical applications in the treatment of periodontal diseases.

### 3.3 Antibacterial efficacy of different NPs with respect to single-species biofilm formation

**Fig. 4:**
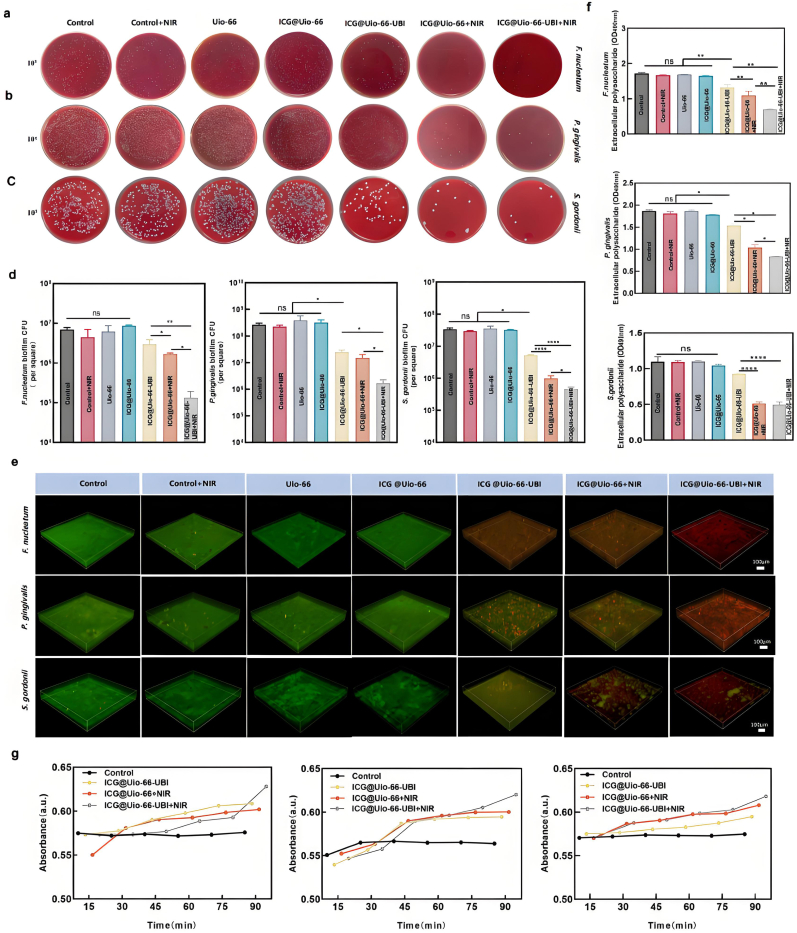
Inhibition effect of ICG@Uio-66-UBI NPs on *F. nucleatum, P. gingivalis and S. gordonii* biofilm formation. The images of (a) *F. nucleatum*, (b) *P. gingivalis* and (c) *S. gordonii clones*. (d) Corresponding statistical data for biofilm colonies of *F. nucleatum, P. gingivalis* and *S. gordonii* biofilms. (e) The 3D live/dead images of 3-day biofilms (dead bacteria, stained red; live bacteria, stained green) for *F. nucleatum, P. gingivalis* and *S. gordonii*. (f) Statistical data for EPS of *F. nucleatum P. gingivalis* and *S. gordonii*. (g) Nucleic acid leakage of *F. nucleatum, P. gingivalis and S. gordonii* in different groups. n = 5,∗p < 0.05, ∗∗p < 0.01, ∗∗∗p < 0.001, ∗∗∗∗p < 0.0001, ns, not significant.

The in vitro antibiofilm efficacy of ICG@Uio-66-UBI NPs was investigated against *F. nucleatum*, *P. gingivalis*, and *S. gordonii*, which are representative periodontal pathogens and porphyrin-producing bacteria, respectively. The investigation was conducted following 808nm NIR irradiation given the significantly greater penetration depth of NIR light [9,35]. We established single-species biofilms and confirmed their successful formation using confocal microscopy (Fig. S2). The results (Fig. 4) illustrated the disruptive impact of various NPs on the formation of *F. nucleatum*, *P. gingivalis*, and *S. gordonii* biofilms. Following treatment, the antibacterial effects of different NPs on single-species biofilms were assessed based on EPS production, CFU counts and biofilm biomass. EPS, which protects cells from external stimuli and stabilizes microbial communities, also promotes microbial aggregation and biofilm formation. As shown in Fig. 4f and S4, the EPS production in biofilms of *F. nucleatum* and *P. gingivalis* was significantly reduced in the ICG@Uio-66-UBI + NIR group compared to the control. However, the EPS production in *S. gordonii* biofilms did not show the most pronounced disruption under the combined action of antimicrobial peptides and aPDT. This resistance may stem from the higher curdlan content in the EPS of *S. gordonii*, a gram-positive bacterium, whose polysaccharide suspension coagulates at high temperatures, enhancing its resistance to antimicrobial agents. Following the disruption of the EPS in the biofilms, the OD260nm values of the three bacterial species in the ICG@Uio-66-UBI + NIR NPs group were significantly higher than those in any other experimental group, as illustrated in Fig. 4g. This observation indicates the most pronounced leakage of DNA and RNA from the bacterial cells. The enhanced antibacterial effect can be attributed to the targeted interaction of cationic UBI29-41 with LPS on *P. gingivalis* and *F. nucleatum* and LTA on *S. gordonii*, altering the permeability of the bacterial cell membrane and causing the leakage of intracellular components, such as nucleic acids and proteins, ultimately leading to bacterial cell death [36]. In the absence of NIR exposure, the growth of single-species biofilms in both the Uio-66 and ICG@Uio-66 groups was similar to that in the control group. When NIR irradiation was provided, the growth of *F. nucleatum*, *P. gingivalis* and *S. gordonii* biofilms was significantly reduced in the ICG@Uio-66-UBI and ICG@Uio-66-UBI-mediated aPDT groups (p < 0.05), with the latter showing a remarkable 2 log reduction approximately in CFU (Fig. 4a–d). Biomass production followed a similar trend to CFU counts (Fig. S5), with the ICG@Uio-66-UBI group exhibiting a significant decline in biomass and CFU values compared to the control (p < 0.01). Additionally, Fig. 4e provides a three-dimensional (3D) reconstruction of 3-day biofilms. 3D live/dead staining images demonstrated that the control group, which did not receive any nanomaterial treatment, exhibited the highest abundance of viable bacteria stained green irrespective of NIR exposure. Meanwhile, Uio-66 and ICG@Uio-66 alone had no significant effect on biofilm formation. In contrast, the combination of aPDT and UBI29-41 in the ICG@Uio-66-UBI group significantly augmented the proportion of dead bacteria within the biofilm. Nevertheless, only partial bacterial cell death was observed in the ICG@Uio-66-UBI group without NIR irradiation and the ICG@Uio-66-mediated aPDT group.

ROS generated through the interaction of photoexcited NPs and light irradiation, play a critical role in eradicating periodontal pathogens by damaging cellular components such as proteins, nucleic acids, and lipids [[37], [38], [39]]. Therefore, the multi-target antibacterial therapy of aPDT makes it challenging for microorganisms to develop resistance. However, gram-negative bacteria like *P. gingivalis* and *F. nucleatum*, with their low-permeability outer membranes, exhibit greater ROS resistance than gram-positive bacteria. In the present study, the overall positive charge of UBI29-41 facilitated the accumulation of NPs on the polyanionic surfaces of microbial cells containing LPS [40]. The electrostatic interaction between UBI29-41 and LPS of *P. gingivalis* and *F. nucleatum* enhanced generation of ROS around the bacteria. TEM revealed that *P. gingivalis* in the control group presented normal morphology, but the bacterial cell wall was ruptured and there was content leakage in the ICG@Uio-66-UBI + NIR group (Fig. S15). This demonstrated that UBI enhanced the antibacterial effects of aPDT. Interestingly, although *S. gordonii* was more ROS-sensitive than *P. gingivalis* and *F. nucleatum*, the findings in the ICG@Uio-66-UBI + NIR group suggest that modulating charge dynamics on gram-positive bacterial cell walls through cationic molecule binding to LTA also plays a pivotal role in inhibiting biofilm formation.

### 3.4 Antibacterial efficacy of different NPs with respect to multi-species biofilm formation

**Fig. 5:**
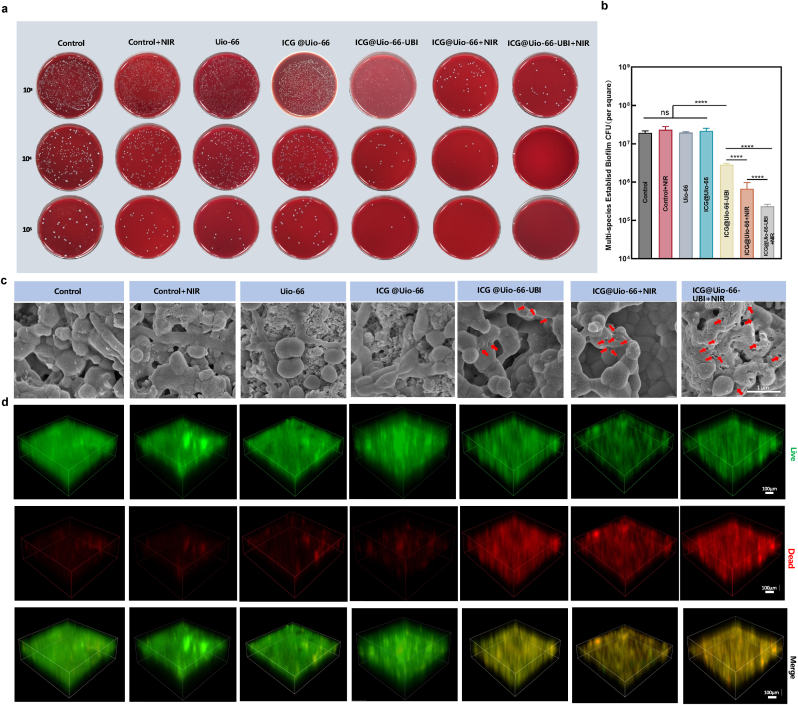
Antibacterial effects on established multi-species biofilms. (a) The images of mixed bacteria *clones*. (b) Corresponding statistical data for multi-species biofilm colonies. (c) TEM images of multi-species following treatment with various nanoparticles (red represent the disruption of the cell membrane.) (d) The 3D live/dead images of 3-day multi-species biofilms (dead bacteria, stained red; live bacteria, stained green). n = 5,∗p < 0.05, ∗∗p < 0.01, ∗∗∗p < 0.001, ∗∗∗∗p < 0.0001, ns, not significant.

The pathogenesis of periodontal disease is not driven by a single pathogen but rather by the synergistic aggregation of multiple bacterial species and the formation of complex mixed biofilms [41]. To evaluate the antibiofilm efficacy of ICG@Uio-66-UBI, we constructed a multi-species biofilm model comprising *F. nucleatum*, *P. gingivalis*, and *S. gordonii*. Quantitative analysis of bacterial viability via colony-forming unit (CFU) counts revealed that ICG@Uio-66-UBI exhibited a more pronounced bactericidal effect against multi-species biofilms compared to single-species biofilms, achieving an approximate 1 log reduction in CFU (Fig. 5a, b). Notably, the combination of ICG@Uio-66-UBI with antimicrobial photodynamic therapy (aPDT) further enhanced this effect, resulting in a remarkable 2 log reduction in CFU.

Scanning electron microscopy (SEM) was employed to assess the morphological changes in the three bacterial species following nanoparticle treatment. As depicted in Fig. 5c, bacteria in the control, control + NIR, Uio-66, and ICG@Uio-66 groups maintained normal morphology. In contrast, bacteria in all treatment groups exhibited varying degrees of surface indentation, with significant biofilm dissipation observed in the ICG@Uio-66-UBI + NIR group. This phenomenon can be attributed to the localized generation of ROS, which induced protein and lipid denaturation, ultimately leading to bacterial membrane degradation.

Further validation was provided by 3D live/dead staining of the multi-species biofilms (Fig. 5d). Compared to the control groups, the ICG@Uio-66-UBI, ICG@Uio-66 + NIR, and ICG@Uio-66-UBI + NIR groups demonstrated a significantly higher proportion of dead bacteria within the biofilm. However, the extent of bacterial death in the multi-species biofilm was less pronounced than that observed in single-species biofilms, even after treatment with ICG@Uio-66-UBI combined with aPDT. This discrepancy may be explained by the unique role of *F. nucleatum* as a bridging bacterium, which facilitates the formation of multi-species biofilms by mediating interactions between *P. gingivalis* and *S. gordonii* [42]. Additionally, multi-species biofilms inherently exhibit enhanced pathogenicity and resistance to antimicrobial agents due to their increased biomass and metabolic activity, potentially accounting for the reduced efficacy of ICG@Uio-66-UBI against these complex communities compared to single-species biofilms.

### 3.5 Antibacterial mechanisms of ICG@Uio-66-UBI

**Fig. 6:**
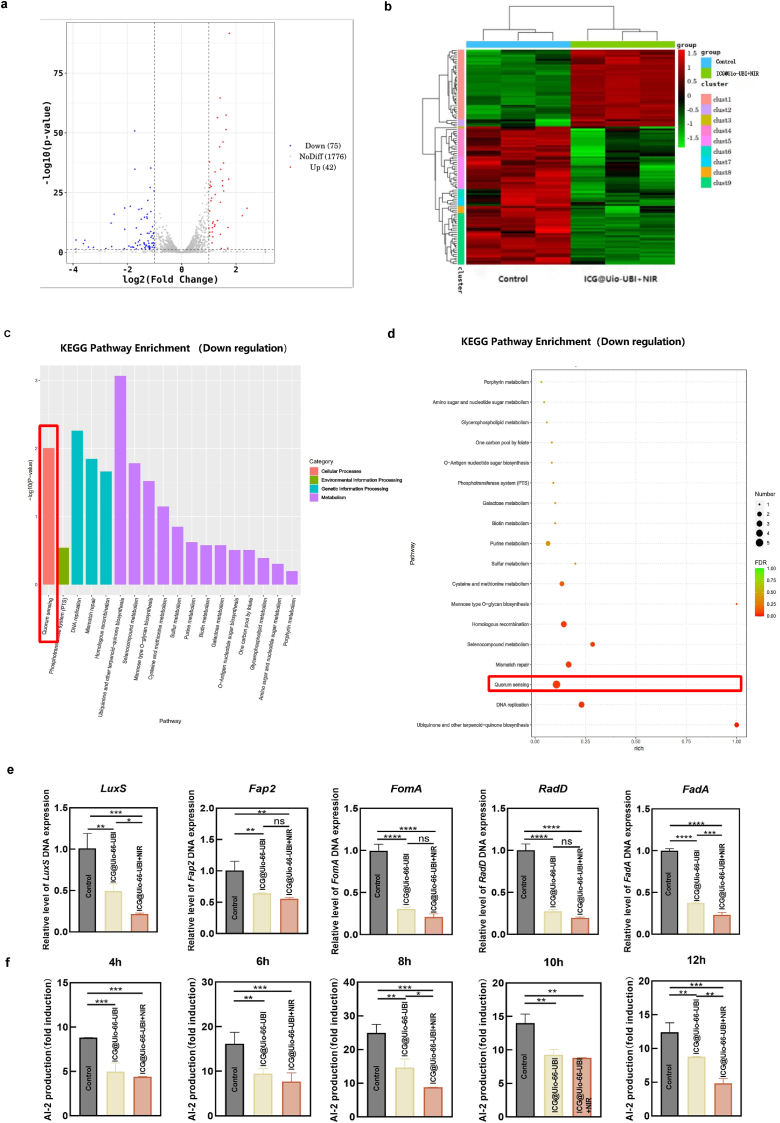
RNA-seq analysis of *F. nucleatum* treated with ICG@Uio-66-UBI+NIR. (a) Volcano plots from RNA-seq analysis depicting differentially expressed genes between the Control and ICG@Uio-66-UBI+NIR groups. (b) Heatmap illustrating the differential expression of genes associated with bacterial biofilm formation. (c, d) KEGG pathways enrichment analysis of downregulated genes. (e) RT-qPCR analysis of the relative mRNA level of virulence factor genes in *F. nucleatum* biofilms. (f) ICG@Uio-66-UBI inhibits secretion of AI-2 signaling molecule by *F. nucleatum.* n = 5,∗p < 0.05, ∗∗p < 0.01, ∗∗∗p < 0.001, ∗∗∗∗p < 0.0001, ns, not significant.

To further explore the anti-biofilm mechanisms induced by ICG@Uio-UBI + NIR treatment, RNA-seq analysis was conducted to identify DEGs in *F. nucleatum* treated with ICG@Uio-UBI + NIR (Fig. 6). As shown in the volcano plots (Fig. 6a), a total of 1893 genes were co-expressed in both the control and ICG@Uio-66-UBI + NIR groups. Meanwhile, 42 genes were up-regulated and 75 genes were down-regulated in *F. nucleatum*. The expression levels of DEGs were analyzed using hierarchical clustering, and robust within-group repeatability was observed. However, there was a significant difference in the expression levels of these genes between the ICG@Uio-66-UBI + NIR group and the control group (Fig. 6b).

Subsequently, KEGG and GO functional enrichment analysis was performed. As depicted in Fig. S6, during GO enrichment analysis, the functions of genes and proteins are typically categorized into three modules: molecular function, biological process, and cellular component. In this study, GO analysis revealed the significant enrichment of DEGs in amino acid metabolic processes, energy metabolism activities, biosynthetic process, and fatty acid metabolism. The amino acid metabolic activities included “nicotinamide nucleotide metabolic” and “nicotinamide nucleotide biosynthetic” processes. Meanwhile, “conversion from NAD to NADP” and “ATP-dependent activity” were related to energy metabolism. Simultaneously, we noted that the GO terms “DNA primase activity” and “transcription regulator activity” were also significantly altered, indicating that ICG@Uio-66-UBI + NIR treatment disrupts protein synthesis-associated processes (Fig. S8a and b). KEGG pathway analysis was further performed to determine the antibacterial mechanisms underlying ICG@Uio-66-UBI, and the enrichment of 18 pathways was identified. As illustrated in Fig. 6c and d, QS pathways were significantly enriched in the KEGG enrichment analyses and were downregulated by ICG@Uio-66-UBI + NIR treatment. The LuxS/AI-2 QS system is a regulatory mechanism present in both Gram-negative and Gram-positive bacteria, with a particularly strong association with periodontal pathogens [43]. During bacterial proliferation, the LuxS protease facilitates the upregulation of autoinducer AI-2 synthesis, thereby modulating the formation of bacterial biofilms. As depicted in Fig. 6e, the gene expression of *LuxS* decreased to 21.6% in the ICG@Uio-66-UBI + NIR group. Meanwhile, as shown in Fig. 6f, ICG@Uio-66-UBI significantly inhibited the production of AI-2 signaling molecules in *F. nucleatum*, and this inhibition increased as time extends. These findings demonstrated that the antibacterial effects of ICG@Uio-66-UBI are likely mediated by its interference with *LuxS* gene expression in the QS system of *F. nucleatum*. This reduces AI-2 secretion and attenuates bacterial virulence to inhibit biofilm formation. These result is consistent with those reported by Jang [44].

To further validate the role of the AI-2 signaling molecule, *F. nucleatum* AI-2 and its inhibitor, D-galactose were employed to investigate formation of *F. nucleatum*, *P. gingivalis*, and *S. gordonii* biofilms, utilizing crystal violet staining and confocal microscopy for assessment. As previously reported, semi-purified *F. nucleatum* AI-2 triggered biofilm formation in periodontopathogens [44], Consistent with this, the addition of purified *F. nucleatum* AI-2 significantly increased biofilm production (p < 0.05) (Fig. S10a, S11a and S12a). Conversely, the addition of D-galactose at varying concentrations (20 mM, 100 mM, and 200 mM) gradually reduced biofilm biomass in all three species, as quantified by crystal violet staining. Notably for *P. gingivalis* and *S. gordonii*, while D-galactose did not significantly suppress AI-2 activity at a concentration of 20 mM, biofilm formation was markedly inhibited by D-galactose at a concentration of 200 mM compared with the 20 mM groups (p < 0.01). Biofilm formation was also visualized through confocal imaging analysis. Compared with the control group, there was a notably elevated proportion of live bacteria following the treatment with ICG@Uio-66-UBI in the presence of *F. nucleatum* AI-2. Nevertheless, D-galactose significantly increased the proportion of dead bacteria within the biofilm of *F. nucleatum, P. gingivalis*, and *S. gordonii* when cultured with *F. nucleatum* AI-2 (Fig. S10c, S11c and S12c). These results indicate that *F. nucleatum* AI-2, regulated by LuxS, is a key signaling molecule affecting the formation of periodontium plaque biofilm. Since QS promotes biofilm formation, it can be considered a potential target against bacterial infection. Additionally, above results also highlighted that ICG@Uio-66-UBI can effectively inhibit biofilm formation by targeting the QS system.

### 3.6 ICG@Uio-66-UBI modulates the LuxS/AI-2 quorum sensing pathway

Studies have shown that as bacterial population density increases, the QS system upregulates AI-2 secretion via LuxS protease, enhancing the expression of genes related to bacterial adhesion and virulence factors, thereby promoting biofilm formation [45]. Key virulence factors of *F. nucleatum*, such as adhesins RadD and FadA, play critical roles in epithelial cell adhesion and invasion [46]. Additionally, virulence factors like heat-modified protein (FomA) and fatty-acid-binding protein 2 (Fap2) are closely associated with plaque biofilm formation [47]. To further explore the impact of the LuxS/AI-2 system on bacterial virulence, qRT-PCR was performed to validate the expression of virulence-associated DEGs (*RadD, FadA, FomA, Fap2*). The results revealed significant downregulation of *RadD, FadA, FomA*, and *Fap2* (p < 0.01) in the ICG@Uio-66-UBI + NIR group (Fig. 6e).

Similarly, *P. gingivalis*, detected in 85.75% of subgingival plaques from chronic periodontitis patients, relies on virulence factors for microbial coaggregation, biofilm formation, and homeostasis [47,48]. In this study, the expression of haemagglutinins (*HagA, HagB*) and virulence factor genes (*RgpA, RgpB, Kgp*) in *P. gingivalis* was evaluated after treatment with ICG@Uio-66-UBI NPs. As shown in Fig. S8a, the mRNA levels of *RgpA, RgpB, HagA, HagB,* and *Kgp* decreased significantly (p < 0.05) in the ICG@Uio-66-UBI + NIR group compared to the control. Notably, these virulence factors exhibited lower sensitivity to ICG@Uio-66-UBI alone than to ICG@Uio-66-UBI + NIR. Additionally, the secretion of AI-2 by *P. gingivalis* followed a similar trend to that of *F. nucleatum* (Fig. S8c).

These findings suggest that the combination of antimicrobial peptides and aPDT effectively inhibits plaque biofilm formation [49]. This is attributed to the neutralization of *P. gingivalis* surface LPS by UBI29-41 and the penetration of ROS generated via aPDT through the bacterial outer membrane/cell wall, damaging DNA encoding virulence and adhesion factors [50]. Collectively, these results demonstrate that ICG@Uio-66-UBI disrupts the LuxS/AI-2 system, modulates related gene expression, and inhibits periodontal plaque biofilm formation.

### 3.7 Immunomodulatory characteristics of ICG@Uio-66-UBI NPs

**Fig. 7:**
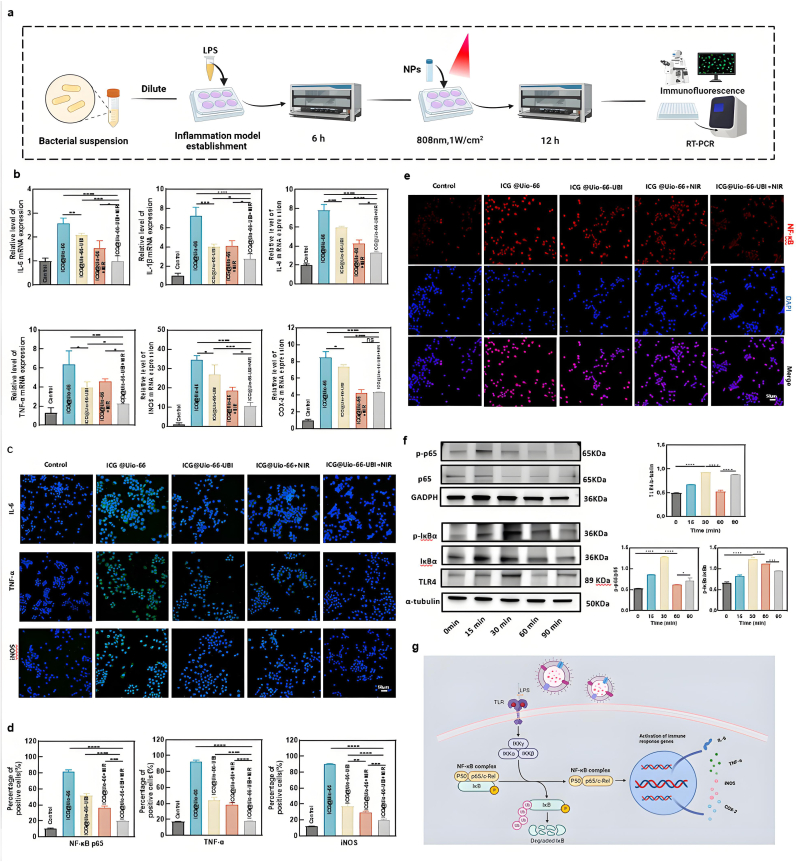
ICG@Uio-66-UBI NPs exhibit inhibitory effects on bacterial-induced macrophage inflammatory response in vitro. (a) The diagram illustration of procedure for establishing an in vitro inflammation model. (b) qRT-PCR analysis of the relative mRNA level of pro-inflammatory cytokines including IL-1β, IL-6, IL-8, TNF-α, iNOS and COX-2 in a treatment model. (c) Immunofluorescence staining of IL-6, TNF-α and iNOS in a treatment model and (d) corresponding analysis of positive cells expression. (e) Immunofluorescence staining was performed to detect alterations in NF-κB/p65 protein levels in Raw 264.7 cells and images collected by CLSM. (f) Western Blot of TLR4, NF-κB/p65 and IκBα, β-actin and GADPH was used as internal control. (g) Schematic representation of the potential mechanis by which ICG@Uio-66-UBI NPs inhibit inflammation. Scale bar: 50 μm. n = 5, ∗p < 0.05, ∗∗p < 0.01, ∗∗∗p < 0.001, ∗∗∗∗p < 0.0001, ns, not significant.

The LPS produced by *Porphyromonas gingivalis* activates monocytes, macrophages, and other immune cells, triggering the release of pro-inflammatory cytokines such as IL-6, IL-1β, IL-8, TNF-α, and COX-2. These cytokines play pivotal roles in periodontal inflammation by inducing B-cell apoptosis and amplifying inflammatory cascades through mutual positive regulation [[51], [52], [53], [54]]. To evaluate the anti-inflammatory potential of our nanoplatform, RAW264.7 cells were stimulated with LPS for 6 h and treated with various nanoparticles (NPs) with or without 808-nm near-infrared (NIR) irradiation (1 W/cm^2^, 10 min). Notably, LPS stimulation significantly upregulated the expression of IL-6, IL-1β, IL-8, iNOS, COX-2, and TNF-α in the inflammatory control group (ICG@Uio-66 group), but this effect was markedly attenuated in the ICG@Uio-66 + NIR group (Fig. 7b). This suppression can be attributed to the ability of aPDT to eliminate periodontal pathogens and inhibit the release of virulence factors, thereby reducing pro-inflammatory cytokine production—a finding consistent with previous reports demonstrating aPDT-mediated inactivation of TNF-α and IL-1β [55]. Furthermore, treatment with ICG@Uio-66-UBI significantly downregulated the expression of these inflammatory factors (p < 0.05), likely due to the electrostatic interaction between the cationic UBI29-41 peptide and anionic LPS, which mitigates LPS-induced macrophage activation [56,57].

In this study, the ICG@Uio-66-UBI + NIR group exhibited the highest inhibition of IL-6, IL-1β, IL-8, iNOS, COX-2, and TNF-α gene expression. In order to further validate these findings, cell-based immunofluorescence experiments were conducted and the protein expression of inflammatory factors was observed across different experimental groups. Immunofluorescence analysis (Fig. 7c, d & S13) revealed the robust protein expression of IL-6, TNF-α, and iNOS in the ICG@Uio-66 group (inflammation group). However, the fluorescence signal for these inflammatory factors was attenuated upon treatment with ICG@Uio-66-UBI combined with 808nm NIR irradiation. This indicated that the expression of these proteins was downregulated in the ICG@Uio-66-UBI + NIR group, reaching levels comparable to those in the control group. This finding corroborated that the concurrent application of the antimicrobial peptide UBI29-41 with aPDT could effectively and synergistically impede the release of inflammatory factors for periodontitis treatment. Subsequently, an immunofluorescence assay was employed to determine the inhibition of the NF-κB pathway by ICG@Uio-66-UBI treatment and thereby assess immune response regulation. As shown in Fig. 7e and S14, the ICG@Uio-66 group displayed robust green fluorescence in the NF-κB/p65 subunit nuclear translocation assay. In contrast, the treatment groups (ICG@Uio-66-UBI, ICG@Uio-66 + NIR, and ICG@Uio-66-UBI + NIR) exhibited significantly fewer positive cells than the inflammation group (p < 0.001). The findings suggested that the aPDT/UBI29-41 nanoplatform could effectively inhibit the translocation of the NF-κB/p65 subunit, leading to the suppression of NF-κB activation and a subsequent reduction in the release of downstream inflammatory factors regulated by NF-κB.

LPS, a pathogen-associated molecular pattern (PAMP), activates TLR4 and initiates the TLR4-NF-κB signaling pathway [58]. UBI29-41 specifically targets the core oligosaccharide and phosphate groups of LPS, disrupting its structural integrity and preventing the formation of the LPS-LBP-TLR4 trimer [59]. This inhibition blocks NF-κB phosphorylation and subsequent pathway activation (Fig. 7g). Time-course experiments revealed that LPS-induced phosphorylation of IκBα and NF-κB/p65 peaked at 15–30 min but was significantly suppressed by ICG@Uio-66-UBI + NIR treatment at 60–90 min (p < 0.005) (Fig. 7f). TLR4 expression followed a similar pattern, further confirming that ICG@Uio-66-UBI inhibits NF-κB signaling by suppressing IκB phosphorylation and preventing NF-κB/Rel complex nuclear translocation, particularly during later stages. Collectively, these results demonstrate that ICG@Uio-66-UBI, under NIR irradiation, effectively inhibits the NF-κB pathway and reduces inflammatory cytokine release, offering a promising strategy for controlling periodontal inflammation.

### 3.8 In vivo therapeutic performance of ICG@Uio-66-UBI for treating periodontal inflammation

**Fig. 8:**
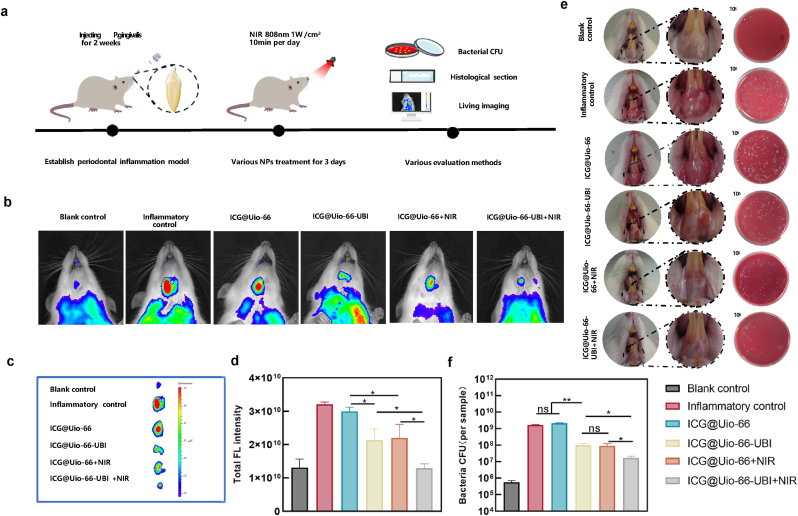
Anti-bacterial and anti-inflammatory capacity of ICG@Uio-66-UBI-mediated aPDT *in vivo*. (a) Schematic illustration of experimental schedule. (b,c) The *in vivo* fluorescence imaging of ROS level at the infection site following treatment with various nanoparticles. (d) The quantification for local ROS level by calculating mean fluorescence intensity ratio. (e) Intraoral photos and colonies of bacteria isolated from gingival tissues were cultured on agar plates. (f) Corresponding statistical data for antibiofilm efficiency. n = 5,∗p < 0.05, ∗∗p < 0.01, ∗∗∗p < 0.001, ∗∗∗∗p < 0.0001, ns, not significant.

To evaluate the antibiofilm and anti-inflammatory efficacy of ICG@Uio-66-UBI, we established a rat model of *P*. *gingivalis*-induced periodontitis (Fig. 8a). Bacterial suspensions were injected into the gingival tissue of rat lower incisors for 14 days to induce inflammation, followed by treatment with various nanocomposites and 808nm NIR irradiation for 3 consecutive days. The treatment effects were visually assessed based on enlarged facial and intraoral photographs of the rats. As shown in Fig. 8e, the gingival tissue was dark red and the presence of swelling was apparent in the inflammatory control group. This indicated the presence of severe localized periodontal inflammation. Compared to the inflammatory control group, the ICG@Uio-66-UBI and ICG@Uio-66+NIR groups exhibited a slight alleviation of gingival inflammation. Meanwhile, the ICG@Uio-66-UBI + NIR group showed a significant improvement in the gingival status, with the gingiva almost appearing normal. As depicted in Fig. 8f, after treatment with different NPs, the CFU counts all showed varying degrees of reduction in different groups when compared with the inflammatory control group. The aforementioned results implied a potential correlation between the mitigation of local inflammation and the antimicrobial activity of the ICG@Uio-66-UBI nanoplatform. Previous studies have demonstrated that UBI29-41 exhibits potent anti-inflammatory effects, thereby promoting wound healing by specifically targeting periodontal pathogens and effectively reducing the bacterial load [60]. Recently, accumulating evidence has also demonstrated that aPDT can also regulate the development of inflammation. Hsing-Wen Sung and colleagues discovered that aPDT can decrease the production of nitric oxide and other byproducts from active macrophages, thereby mitigating the inflammatory response and minimizing damage to local tissues [61]. Therefore, CAMPs may promote the effects of aPDT by targeting pathogenic bacteria to alleviate inflammation, thus achieving rapid and effective treatment.

To validate the production of ROS by the nanoplatform upon NIR irradiation *in vivo*, we conducted live imaging experiments in mice. As shown in Fig. 8b, the inflammatory control group and ICG@Uio-66 group (no NIR irradiation) exhibited significantly elevated fluorescence intensities, which were attributed to the excessive production of ROS due to localized periodontal inflammation caused by bacteria infection. Meanwhile, the attenuated fluorescence intensity in the ICG@Uio-66 + NIR group could be attributed to the rapid bactericidal effect of aPDT. However, the anti-inflammatory effect was suboptimal in this group since the generation of ROS following aPDT treatment did not have any other inherent anti-inflammatory components. In contrast, the fluorescence intensity was markedly reduced in the ICG@Uio-66-UBI + NIR group due to the multimodal therapeutic effect of aPDT/UBI29-41. In this group, the level of ROS was equivalent to that of the control group. Quantitative analysis (Fig. 8,c d) provided statistical evidence supporting the aforementioned graphical findings.

**Fig. 9:**
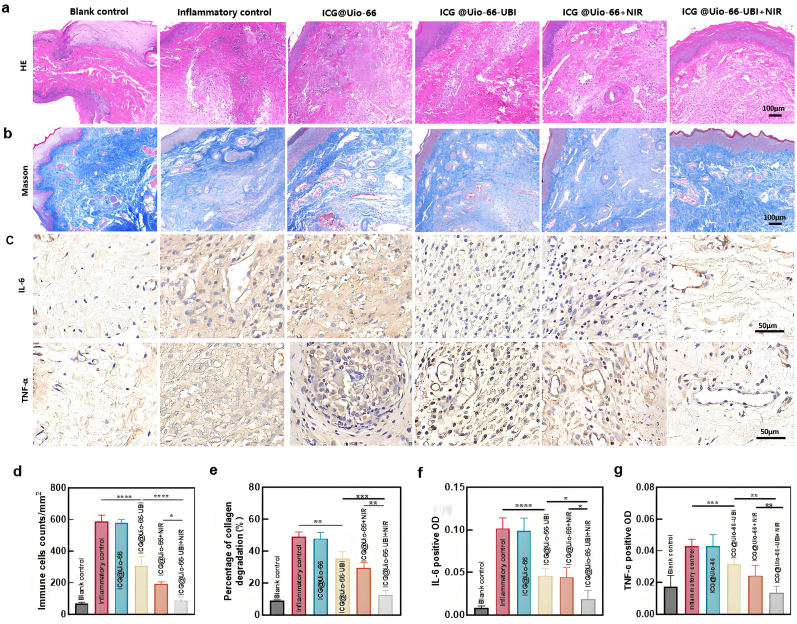
Evaluations of the anti-inflammatory efficacy following different NPs treatments under 808 nm NIR irradiation *in vivo*. (a) Images of H&E staining and (d) The statistical analysis of immune cell counts at the inflammatory site in gingival tissue. (b) Images of Masson staining and (e) The statistical analysis of collagen degradation in gingival tissue. (c) IHC staining images of gingival tissues including IL-6 and TNF-α. (f,g) Quantitation of IL-6, TNF-α levels in IHC staining samples. n = 5, ∗p < 0.05, ∗∗p < 0.01, ∗∗∗p < 0.001, ∗∗∗∗p < 0.0001, ns, not significant.

Then, an *in vivo* study was conducted to further corroborate the efficacy of intervention with ICG@Uio-66-UBI NPs with respect to immunoregulation (Fig. 9). The amount of inflammatory cells in gingival tissues was analyzed through H&E staining. Fig. 9a shows that the inflammatory control group and ICG@Uio-66 group exhibited a substantial infiltration of inflammatory cells in the gingival tissue and a significant reduction in fibroblast density. However, the ICG@Uio-66-UBI (no irradiation) and ICG@Uio-66 + NIR groups exhibited a remarkable reduction in inflammatory cells. Notably, the gingival tissue in the ICG@Uio-66-UBI + NIR irradiation group exhibited a resemblance to healthy tissue. This demonstrated the significant inhibitory effect of combined aPDT and antimicrobial peptide treatment on local inflammation. The statistical analysis of the number of inflammatory cells is presented in Fig. 9d and reaffirms the aforementioned findings.

Collagen integrity, a critical indicator of periodontal health, was evaluated using Masson's trichrome staining (Fig. 9b,e). In the blank control group, collagen fibrils appeared densely packed and well-organized, as indicated by intense blue staining. In contrast, the inflammatory control and ICG@Uio-66 (no NIR) groups exhibited significant collagen degradation, reflecting advanced periodontal damage. Treatment with ICG@Uio-66-UBI or ICG@Uio-66+NIR markedly reduced collagen dissolution, with the ICG@Uio-66-UBI+NIR group showing the most pronounced preservation of collagen structure. Type I collagen, the primary component of periodontal ligaments, is a key biomarker of periodontal disease progression [62]. Pathogen-associated molecular patterns (PAMPs) trigger inflammatory responses, upregulating matrix metalloproteinases (MMPs) in fibroblasts and epithelial cells, which degrade collagen fibers [63]. However, the ROS generated by aPDT not only enhance collagen maturation but also inhibit osteoclast formation, counteracting tissue destruction [64]. The targeted delivery of ROS by UBI29-41 further amplifies these effects, localizing therapeutic action to pathogenic bacteria and minimizing collateral tissue damage. These findings underscore the dual role of ICG@Uio-66-UBI in preserving collagen integrity and mitigating inflammation, highlighting its potential to disrupt the vicious cycle of infection and tissue degradation.

Pro-inflammatory cytokines, particularly IL-6 and TNF-α, play pivotal roles in periodontal pathogenesis. Gingipains and fimbriae from Porphyromonas gingivalis upregulate IL-1β, IL-8, IL-6, and TNF-α, exacerbating tissue destruction [65]. IL-6 promotes collagen degradation, osteoclast activation, and bone resorption, while TNF-α amplifies inflammation by stimulating IL-1 and IL-6 production, further enhancing MMP activity and inhibiting collagen synthesis [66]. Immunohistochemical staining revealed strong expression of IL-6 and TNF-α in the inflammatory control and ICG@Uio-66 (no NIR) groups, as indicated by brownish-yellow staining (Fig. 9c,f ＆g). In contrast, the ICG@Uio-66-UBI + NIR group exhibited minimal immunostaining (p < 0.01), confirming the nanoplatform's ability to suppress pro-inflammatory cytokine production. This reduction in IL-6 and TNF-α levels correlates with decreased collagen degradation and inflammation, demonstrating the therapeutic efficacy of ICG@Uio-66-UBI.

## 4 Conclusions

This study developed ICG@Uio-66-UBI, a NIR light-responsive nanoplatform for precision treatment of periodontal infections. The UBI29-41-modified system enhances targeted ICG delivery to pathogens and synergizes with photodynamic therapy (PDT) to achieve dual antimicrobial and anti-inflammatory effects. UBI29-41 binds selectively to LPS and LTA on bacterial surfaces, amplifying localized singlet oxygen (^1^O_2_) production under NIR irradiation to disrupt biofilms and inhibit plaque formation. Simultaneously, the platform suppresses LPS-TLR4 interactions and NF-κB activation, reducing inflammation and tissue damage. *In vivo* studies confirmed its therapeutic potential, showing reduced inflammatory cell infiltration, inhibited collagen degradation, and downregulated inflammatory mediators. Transcriptome analysis revealed downregulation of biofilm-related genes and the LuxS/AI-2 QS pathway, providing mechanistic insights.

In summary, ICG@Uio-66-UBI offers a novel, dual-action strategy for periodontitis treatment, combining biofilm inhibition with inflammation control, and holds significant clinical promise.

## CRediT authorship contribution statement

**Wen Li:** Writing – original draft, Funding acquisition, Data curation. **Fengqun You:** Formal analysis. **Jie Yang:** Methodology, Investigation. **Deao Gu:** Supervision, Formal analysis. **Yuyang Li:** Supervision, Project administration. **Xuan Zhang:** Visualization, Project administration. **Leiying Miao:** Visualization, Funding acquisition. **Weibin Sun:** Conceptualization.

## Declarations ethics approval and consent to participate

Animal experiments was approved by the Ethical Review Board at Nanjing University (No. #IACUC-D2304013). Human clinical experiments was approved by the Ethical Review Board at Nanjing University (No.NJSH-202023NL-43). All respondents were informed comprehensively and provided written informed consent.

## Data availability

The data are available from the corresponding author on reasonable request.

## Funding

This work was supported by the Natural Science Foundation of Jiangsu under Grant (No.BK20221177), “2015” Cultivation Program for Reserve Talents for Academic Leaders of Nanjing Stomatological School Medical School of Nanjing Univeristy (No.0223A203), Postgraduate Research & Practice Innovation Program of 10.13039/501100002949Jiangsu Province (SJCX▁240009), the 10.13039/100006180Key Project supported by Medical Science and Technology Development Foundation, Nanjing Department of Healthunder Grant (No.ZKX24056), the General Project supported by Medical Science and Technology Development Foundation, Nanjing Department of Healthunder Grant (No.YKK23183,YKK24198), High-Level Hospital Construction Project of Naniing Stomatological Hospital, Affiliated Hospital of Medical School, Institute of Stomatology, Nanjing University (No.0224C051, 0224C020, 0224C006).

## Declaration of competing interest

The authors declare that they have no known competing financial interests or personal relationships that could have appeared to influence the work reported in this paper.

## Data Availability

No data was used for the research described in the article.
