# Supplementary material for: Antimicrobial peptide-targeted photodynamic therapy for preventing periodontal plaque biofilm formation through the disruption of quorum sensing system
Source: Mater Today Bio. 2025 Jun 18;33:101970. doi: 10.1016/j.mtbio.2025.101970 (PMC12268586; doi:10.1016/j.mtbio.2025.101970)
Supplement: Multimedia component 1 [file mmc1.docx]

1. **Supplementary Methods**

***In vitro* photothermal effects**

To measure in vitro photothermal effects, ICG@Uio-66-UBI was placed in plastic tubes and then was irradiated by an 808 nm laser (BWT Beijing Ltd., DS3–11313–0411, max: 5 W, 10,000 Hz, 45μs) with different power densities (0.5 W cm^−2^ , 1.0W cm^−2^ and 2 W cm^−2^) for 10 min. Photothermal images were collected by using an infrared thermal imaging camera (FLIR C7200 C3, USA) every 2mins.

**Live/dead fluorescent staining of Bacterial biofilm**

Biofilm were washed with phosphate buffer solution to rinse out non-adherent bacteria. Each sample were stained with 300 μL of physiological saline, 0.3 μL of SYTO9 and 0.3 μL of PI per well for 15 min under light-proof conditions according to the operating instructions. Biofilm 3D images were collected with CLSM.

**Determination of biofilm biomass**

Biofilm biomass was tested through Crystalline violet staining method. Each hydroxyapatite slices was transferred to a new 24-well plate, biofilm was stained with 0.1% crystalline violet for 15 min after paraformaldehyde fixed it for 20 min Then the biofilms were washed with PBS and decolorization was performed with ethanol (95% v/v). The ethanol was transferred to a 96-well plate. The value of biofilm biomass was determined by evaluating the absorbance at OD_600nm_ with an enzyme meter.

**Extracellular polysaccharide (EPS) measurement of Bacterial biofilm**

Biofilm were washed with phosphate buffer solution to rinse out non-adherent bacteria. Bacteria EPS was tested through the phenol-sulfuric acid method. Briefly, washing with distilled water (40 μL), 6% phenol solution (40 μL diluted in distilled water), and sulfuric acid (97%) after air-drying for 20 min. Then, the plate was incubated for 30 min at room temperature. The quantity of polysaccharides in the biofilm was determined by measuring the absorbance at OD _490nm_.

**Standard plate counting assays**

Bacterial biofilm on hydroxyapatite slices were transferred to EP tubes containing 1 ml medium for suspension, and vortex mixer was used to shake down the biofilm. The biofilm suspension was cultured on the blood agar plates through gradient dilution method and incubated at 37°C for 48h for CFU counting.

**Scanning electron microscopic (SEM) analysis**

The biofilm were rinsed with PBS to eliminate non-adherent bacteria and were fixed in a 2.5% glutaraldehyde for 1-4 hours subsequently. This was followed by gradient ethanol dehydration (30% to 100%). After completely drying, the samples were coated with gold via spraying technique.

**Purification of *F. nucleatum* AI-2**

The AI-2 of *F. nucleatum* was partially purified as described previously (Jang et al., 2013). Briefly, bacteria from an overnight culture of *F. nucleatum* and *V. harveyi* BB170 were diluted 1:20 with fresh culture medium and cultured at 37°C and 30°C, respectively, until the late exponential phase. The culture supernatants were collected by centrifugation at 10,000×g at 4°C. After the culture supernatants were passed through 0.2-μm pore-size membrane filters (Sartorius Stedium Biotech), the filtrate was subsequently passed through a Centricon YM-3 3-kDa exclusion filter (Millipore) and then chromatographed on a C18 Sep-Pak reverse-phase column (Waters Co.)

**Effect of D-galactose on biofilm formation of periodontopathogens**

Bacterial suspensions were added to 24-well plates in the presence of *F. nucleatum* AI-2 (10% vol/vol) and/or sugars at various concentrations and were incubated for 24–48 h under anaerobic conditions. The initial numbers of *F. nucleatum*, *P. gingivalis*, and *S.gordonii* were 1 × 10^8^/ml. The samples were divided into three groups: (1) Control group: The (*F. nucleatum*/*P. gingivalis*/*S.gordonii* )suspension was cultured in a ICG@Uio-66-UBI-free medium; (2) ICG@Uio-66-UBI+NIR group: The (*F.nucleatum*/*P. gingivalis*/*S.gordonii*) suspension and 30 μg/mL ICG@Uio-66-UBI NPs were incorporated into the medium and exposed to NIR irradiation (1 W/cm^2^, 10 min); (3) ICG@Uio-66-UBI+NIR+AI-2 group: The (*F.nucleatum*/*P.gingivalis*/*S.gordonii*) suspension, AI-2 and 30 μg/mL ICG@Uio-66-UBI NPs were incorporated into the medium and exposed to NIR irradiation (1 W/cm^2^, 10 min); (4) ICG@Uio-66-UBI+NIR+AI-2+20mM *D-gal* group: The (*F.nucleatum*/*P.gingivalis*/*S.gordonii*) suspension, AI-2, 20mM *D-gal* and 30 μg/mL ICG@Uio-66-UBI NPs were incorporated into the medium and exposed to NIR irradiation (1 W/cm^2^, 10 min); (5) ICG@Uio-66-UBI+NIR+AI-2+100mM *D-gal* group: The (*F.nucleatum*/*P.gingivalis*/*S.gordonii*) suspension, AI-2, 100mM *D-gal* and 30 μg/mL ICG@Uio-66-UBI NPs were incorporated into the medium and exposed to NIR irradiation (1 W/cm^2^, 10 min); (6) ICG@Uio-66-UBI+NIR+AI-2+200mM *D-gal* group: The (*F.nucleatum*/*P.gingivalis*/*S.gordonii*) suspension, AI-2, 200mM *D-gal* and 30 μg/mL ICG@Uio-66-UBI NPs were incorporated into the medium and exposed to NIR irradiation (1 W/cm^2^, 10 min); Biofilm formation assays were conducted using crystal violet staining and analyzed by confocal laser scanning microscopy, as detailed in previous studies.

**2.Supporting Tables**

**Table S1.** Oligonucleotide primers used in fluorescent *in situ* hybridization

| **Probes** | **Nucleotide Sequence (5'to3')** |
| --- | --- |
| *S. gordonii* ATCC10558 | FITC-5′-ACTGTGCGTTCTACTTGC-3′ |
| *P.gingivalis*ATCC33277 | Cy3 (Cyanine 3)-5′-GTTTTCACCATCMGTCATC-3′ |
| *F.nucleatum*ATCC10953 | DEA (diethylaminocoumarin)-5′-CTTGTAGTTCCGCYTACCTC-3′ |

**Table S2.** Primer sequences used in the antibacterial experiment

| **Gene** | **Forward Sequence (5' to 3')** | **Reverse Sequence (5' to 3')** |
| --- | --- | --- |
| 16srRNA | AATGTAGATGACTGATGGTGAA | TGCGCTCGTTATGGCACTTAA |
| hagA | ACAGCATCAGCCGATATTCC | CGAATTCATTGCCACCTTCT |
| hagB | TGTCGCACGGCAAATATCGCTAAAC | CTGGCTGTCCTCGTCGAAAGCATAC |
| Kgp | AGCTGACAAAGGTGGAGACCAAAGG | TGTGGCATGAGTTTTTCGGAACCGT |
| RgpA | CTGCGAGCGGTATTAGTGGT | CTACCAGCCCGTTTCCAACT |
| RgpB  LuxS(*Fn*)  Fap2  FomA  RadD  FadA  LuxS (*Sg*)  gtfG  rgg | TCGGGACAAGTGTACGAACG  TGCCACATTCCACAGCAGAA  CCAACCCCAACACTTTCATC  GGAATTCCCAACAACTCCACTATTATGTCC  GAAGAAAGAGCACAAGCTGA  GCTTGAAGTCTTTGAGCTCT  CAGGACTCCATACCATTGAACA  CGGATGATGCTAATCAAGTGACC  CTGTTGCCCAGCTGTC | AACCAGTCTTGGGCTTCTCC  CCTATGGGTTGCAGAACAGGT  CCAACCCCAACACTTTCATC  GGAATTCCCAACAACTCCACTATTATGTCC  GAAGAAAGAGCACAAGCTGA  GCTTGAAGTCTTTGAGCTCT  GCGATCTCTTCGAGGCATGAT  CCAACCCCAACACTTTCATC  CCGGTCATAGAGGTCTGA |

**Table S3.** Primer sequences used in this study

| **Gene** | **Forward Sequence (5' to 3')** | **Reverse Sequence (5' to 3')** |
| --- | --- | --- |
| β-actin | CATCCGTAAAGACCTCTATGCCAAC | ATGGAGCCACCGATCCACA |
| IL-1β | TCCAGGATGAGGACATGAGCAC | GAACGTCACACACCAGCAGGTTA |
| IL-6 | CCACTTCACAAGTCGGAGGCTTA | CCAGTTTGGTAGCATCCATCATTTC |
| iNOS  COX-2 | CGGCAAACATGACTTCAGGC  TTCCAATCCATGTCAAAACCGT | GCACATCAAAGCGGCCATAG  AGTCCGGGTACAGTCACACTT |
| TNF-α | ACTCCAGGCGGTGCCTATGT | GTGAGGGTCTGGGCCATAGAA |
