# Supplementary material for: Antimicrobial peptide-targeted photodynamic therapy for preventing periodontal plaque biofilm formation through the disruption of quorum sensing system
Source: Mater Today Bio. 2025 Jun 18;33:101970. doi: 10.1016/j.mtbio.2025.101970 (PMC12268586; doi:10.1016/j.mtbio.2025.101970)
Supplement: Multimedia component 2 [file mmc2.docx]

3.Supplementary Figures

**a b**


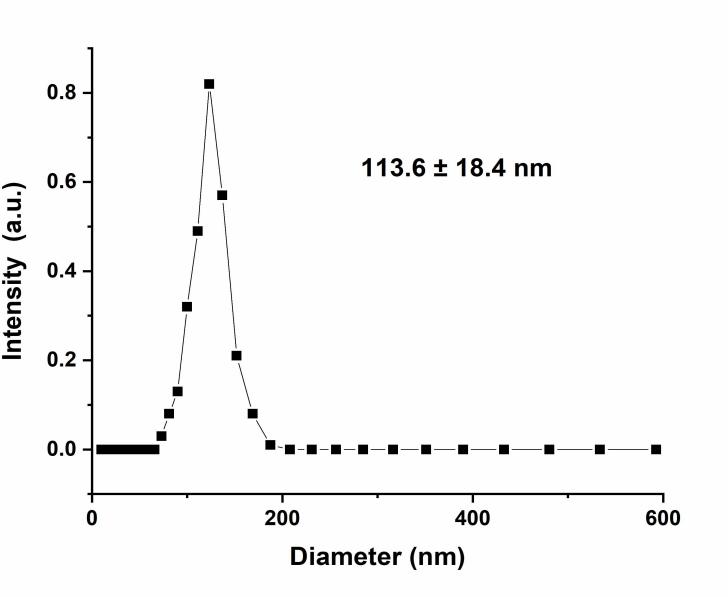

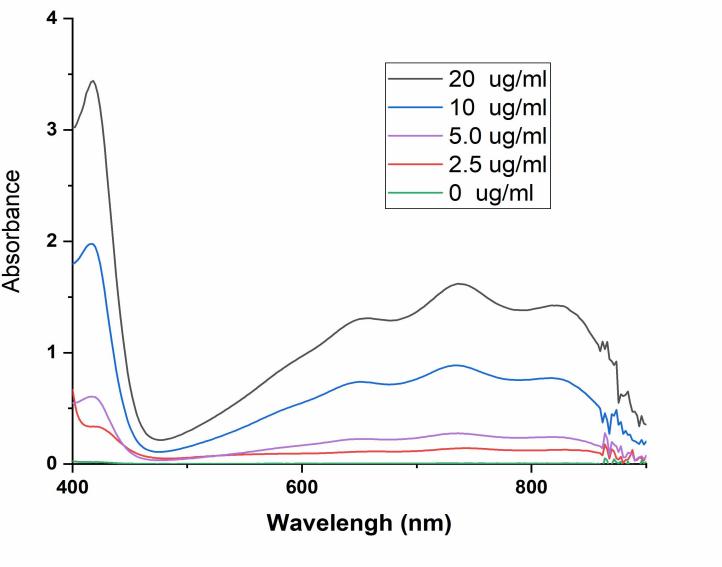


**C d**


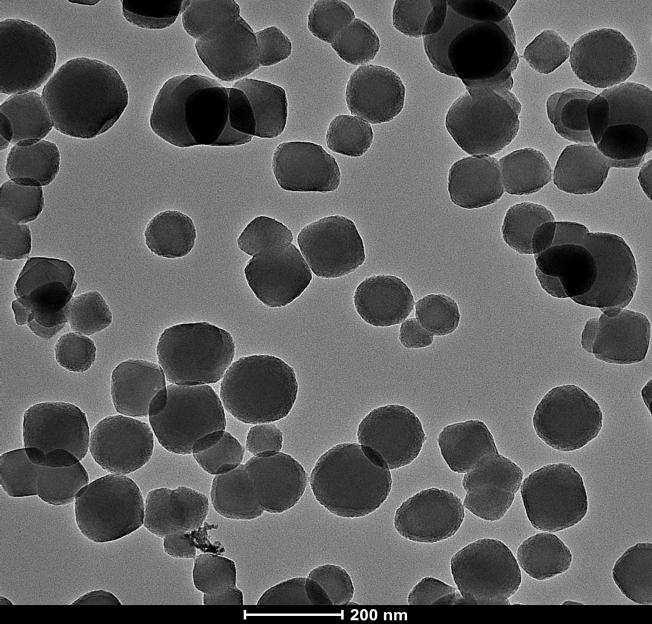

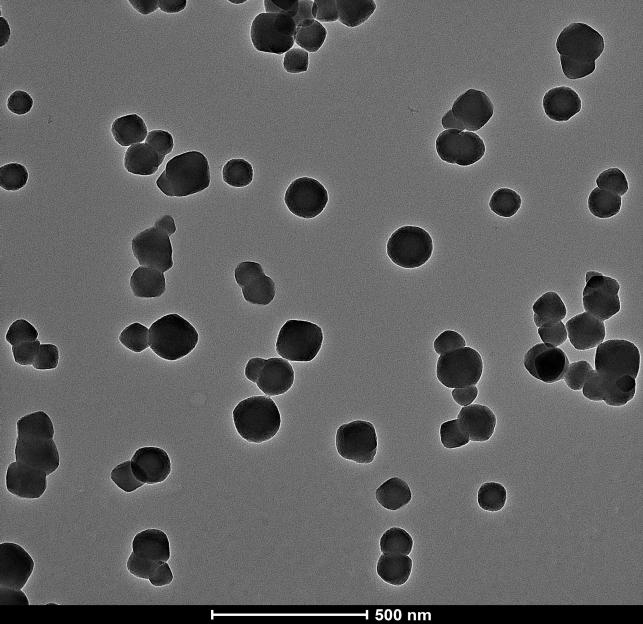


**Fig. S1.** Characterization of ICG@Uio-66 and ICG@Uio-66-UBI NPs. (a) The size distribution of ICG@Uio-

66. (b) UV–vis spectra of different concentrations of ICG@Uio-66 mixed with TMB NPs. (c, d) TEM images of ICG@Uio-66-UBI NPs.

#
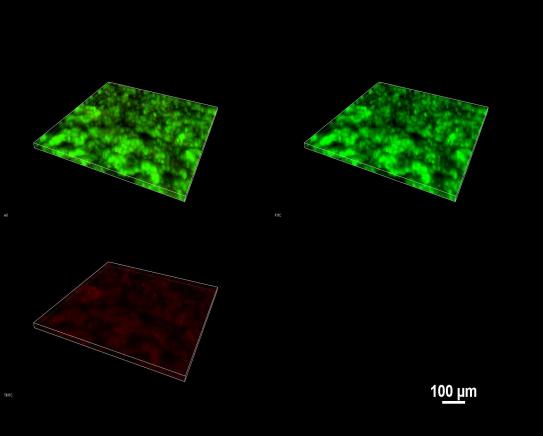

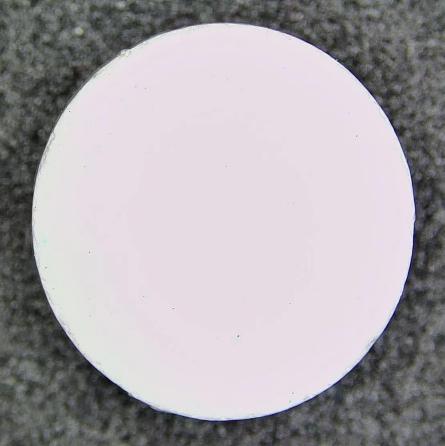

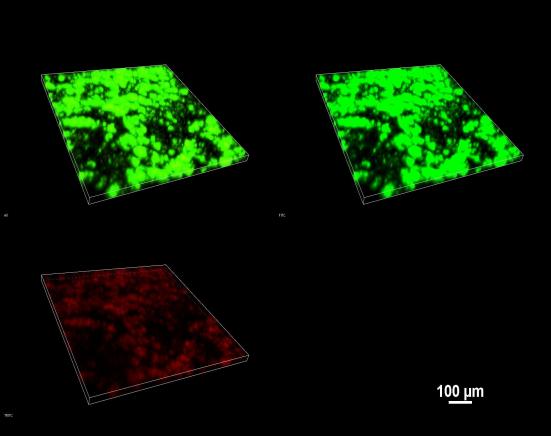
a b C

**Fig. S2.** Single-species biofilms were established on Hydroxyapatite slices. (a,c) Live/ dead staining of *pg* biofilms formed on Hydroxyapatite slices by laser confocal microscopy. (b) Morphology of Hydroxyapatite slices under electron microscope.


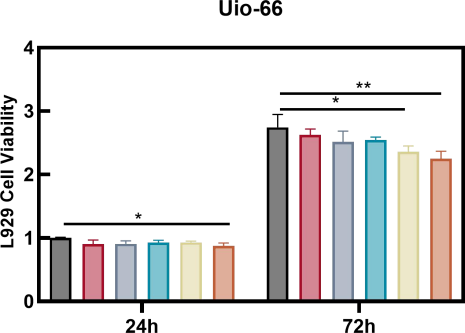

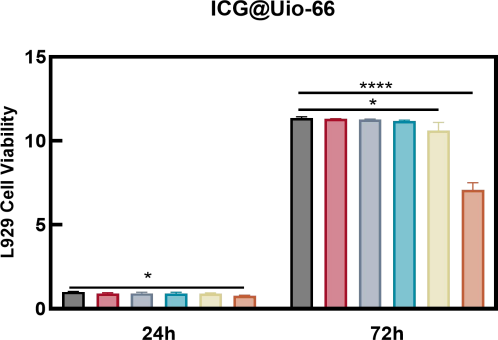

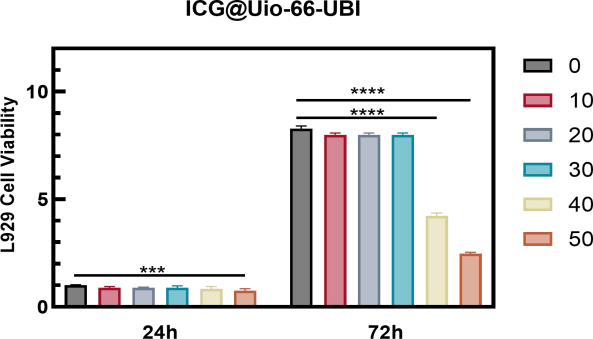
**a b c**

**lung**

**spleen**


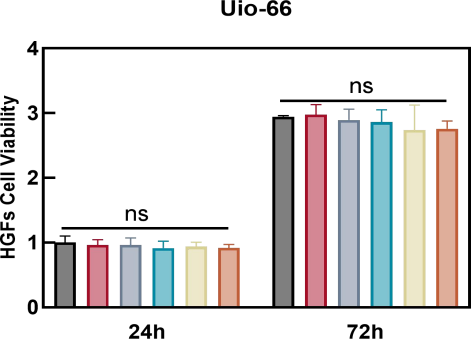

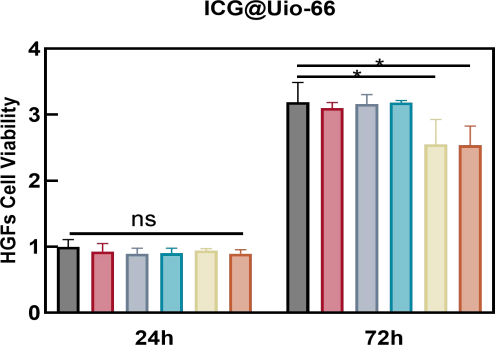

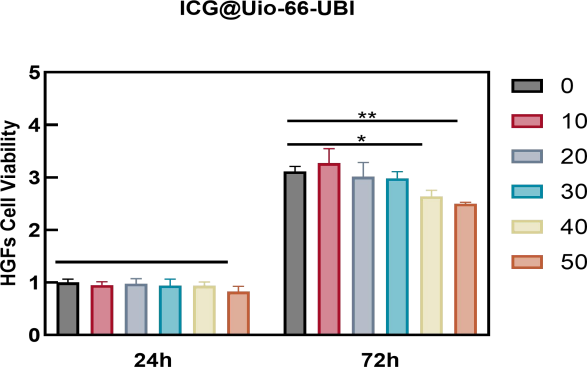
**d e f**

**g**

**Blank control Inflammatory control ICG@Uio-66 ICG @Uio-66-UBI ICG@Uio-66+NIR ICG@Uio-66-UBI+NIR**


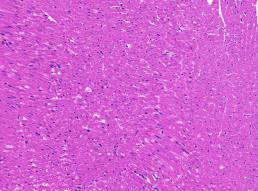

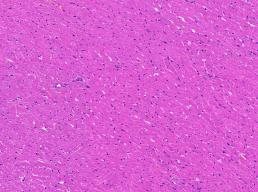

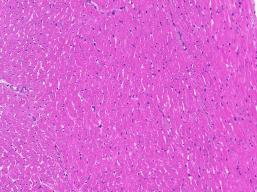

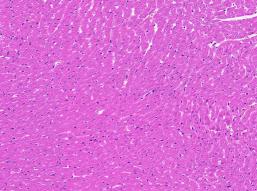

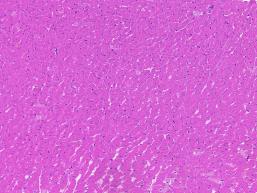

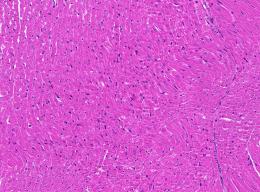

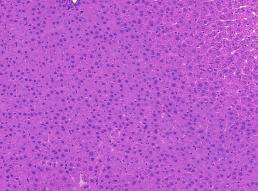

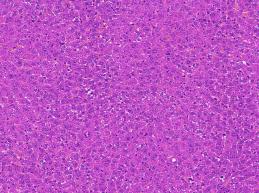

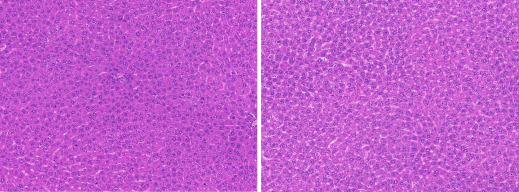

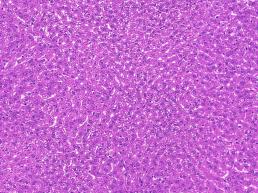

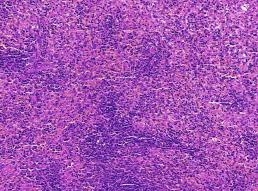

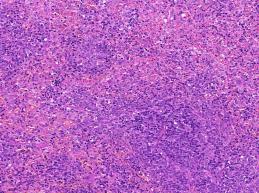

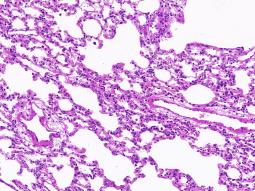

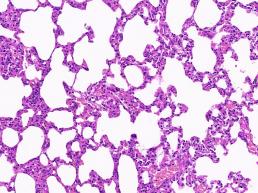

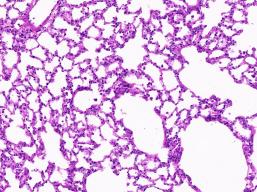

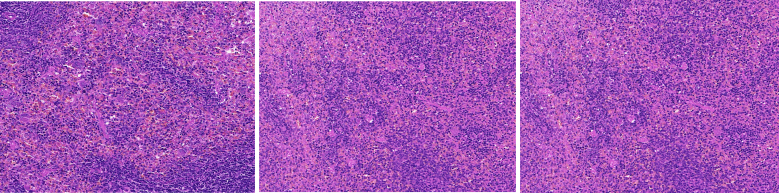

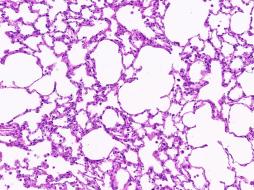

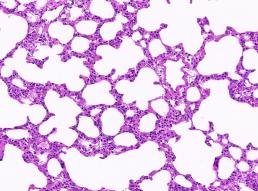

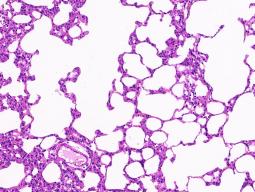

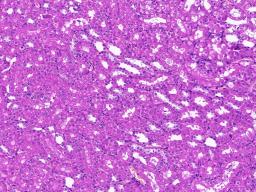

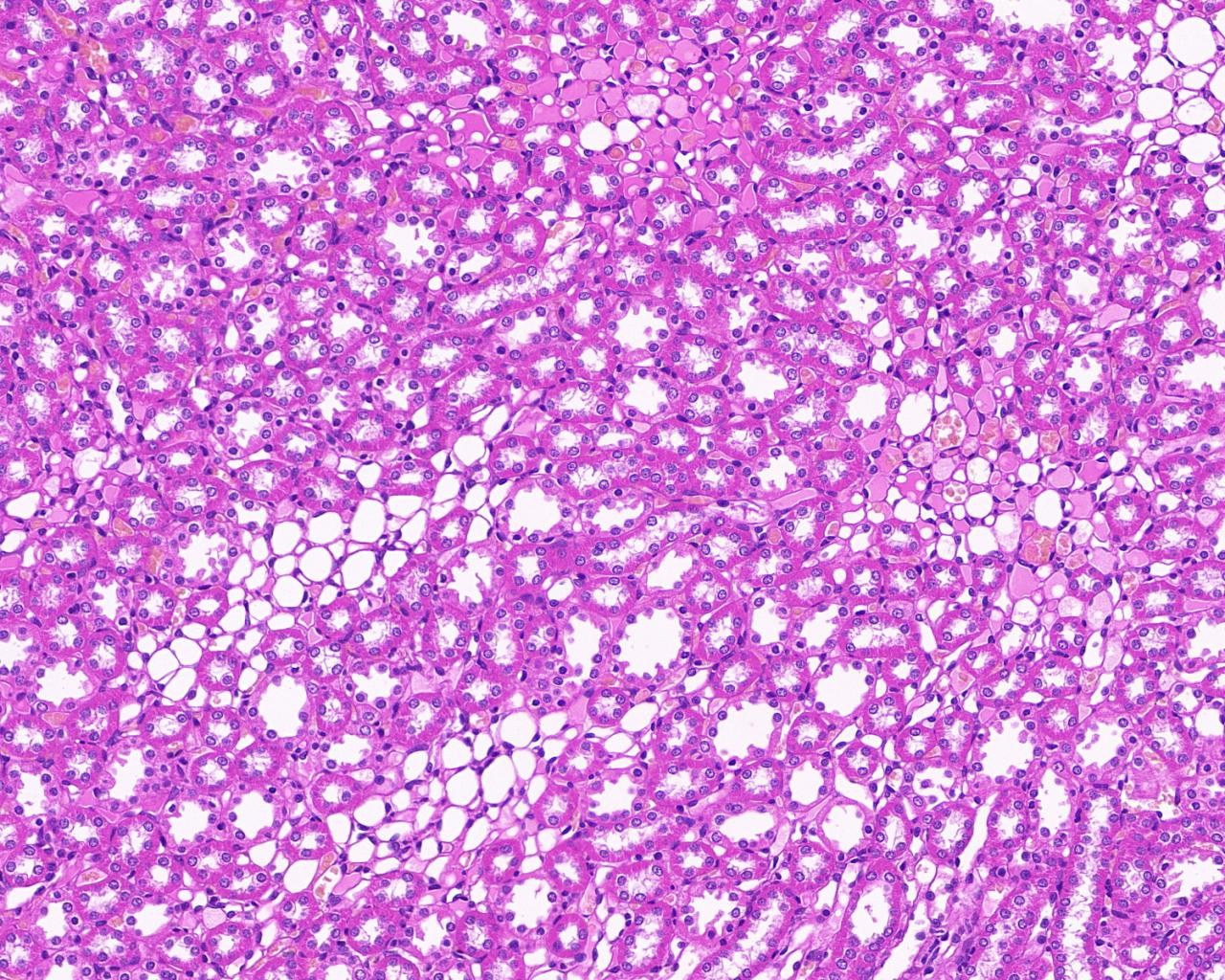

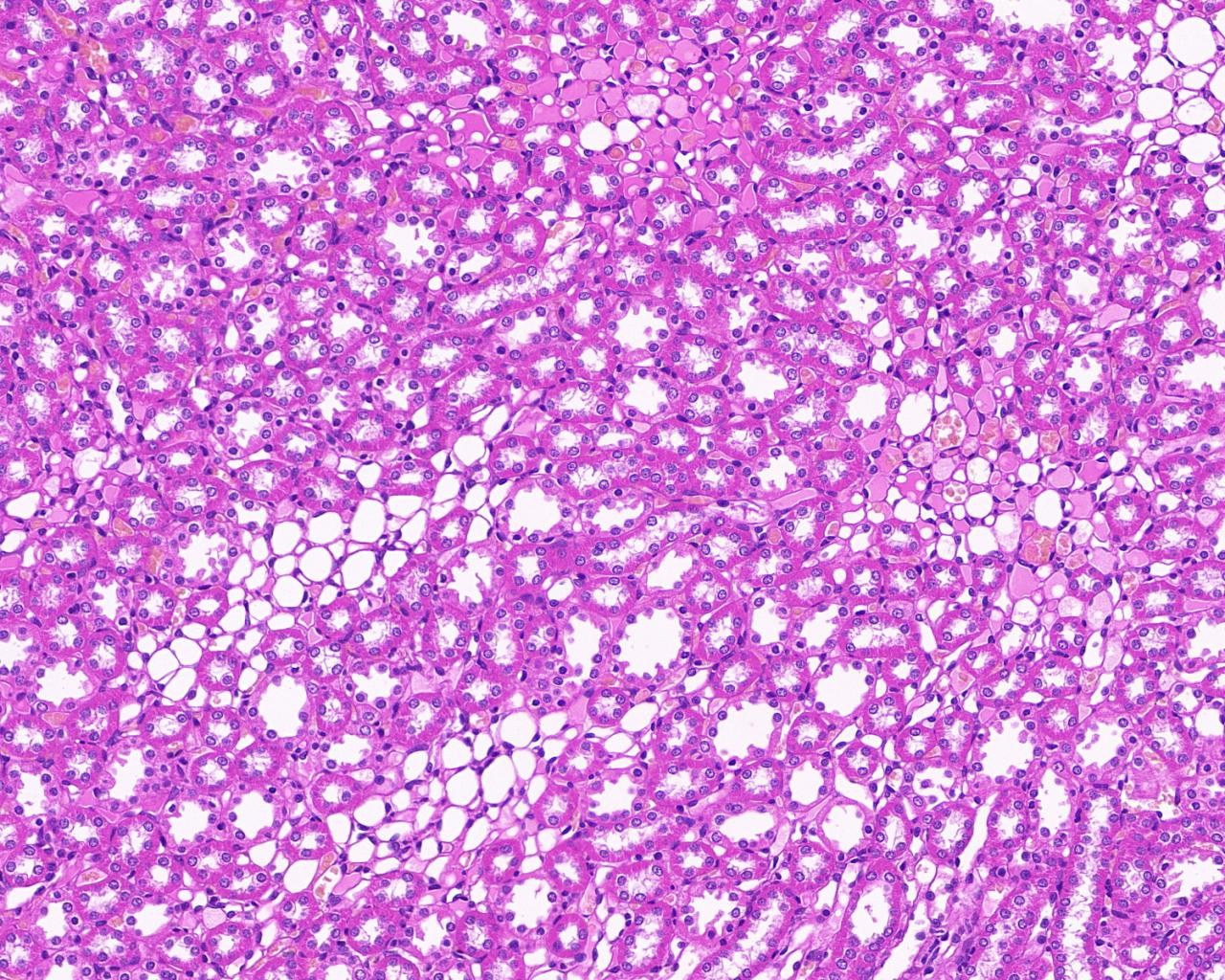

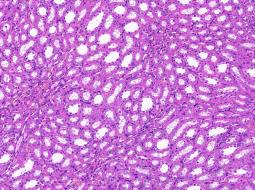

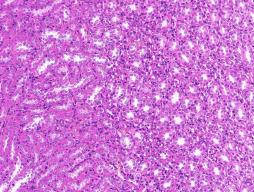

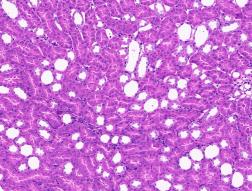

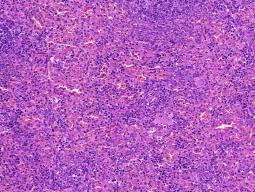

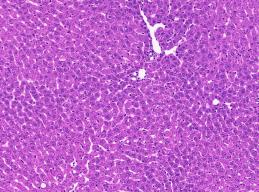


**heart**

**liver**

**Fig. S3.** Biosafety assays of NPs. (a-c) The cell viability of L929 fibroblasts incubated with different concentration (including 0, 10, 20, 30, 40 and 50 μg/mL) of Uio-66, ICG@Uio-66 and ICG@Uio-66-UBI NPs for 24h and 72 h. (d-f) The cell viability of HGFs in different concentration (including 0, 10, 20, 30, 40 and 50 μg/mL) of Uio-66, ICG@Uio-66 and ICG@Uio-66-UBI NPs for 24h and 72 h. (g) H&E staining of major organs (including heart, liver, lung, spleen, and kidney) after various treatments. Scale bar, 100 μm. Statistical significances were calculated via one-way ANOVA. n=3, *p < 0.05, **p < 0.01, ***p < 0.001, ****p < 0.0001, ns, not significant.

**kidney**

# a b


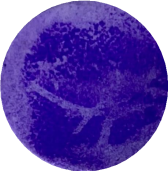

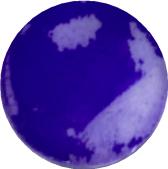

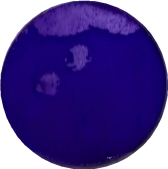

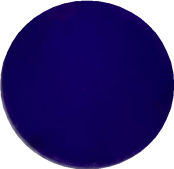

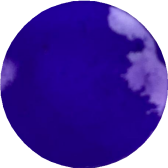

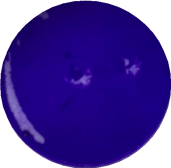

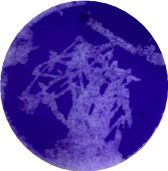


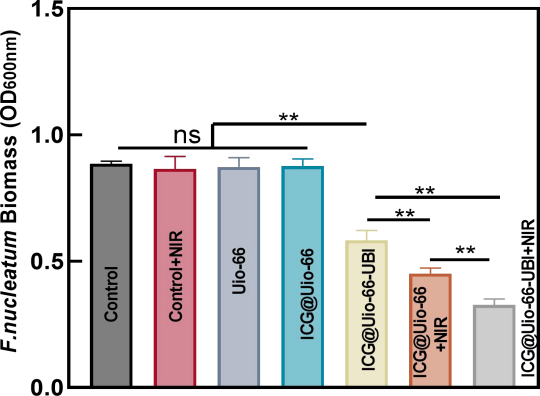
**Control Control+NIR Uio-66 ICG@Uio-66**


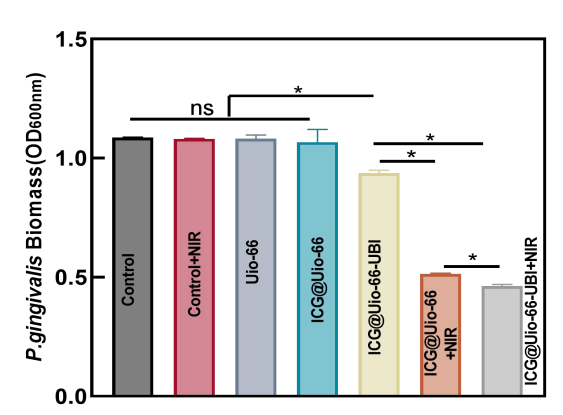
**C d**

**ICG@Uio-66-UBI**

**ICG@Uio-66+NIR**

**ICG@Uio-66-UBI+NIR**


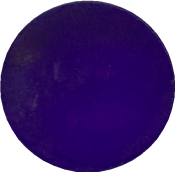


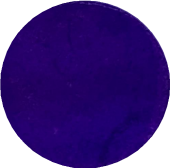

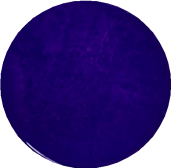

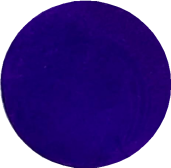

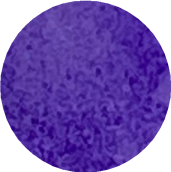

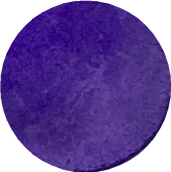

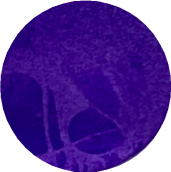


**Control Control+NIR Uio-66 ICG@Uio-66 ICG@Uio-**

**66-UBI**

#
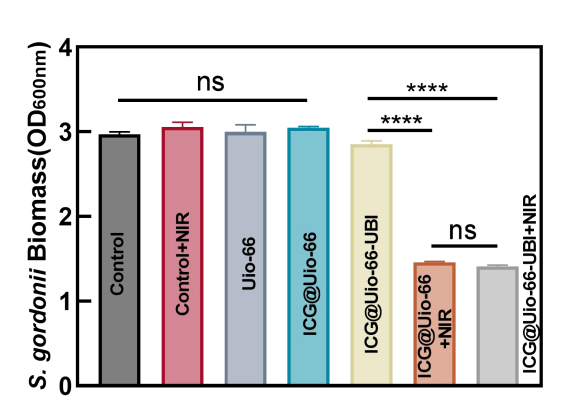
e f

**ICG@Uio-66+NIR**

**ICG@Uio-66-UBI+NIR**


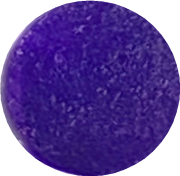

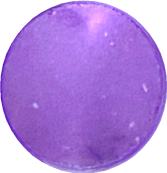


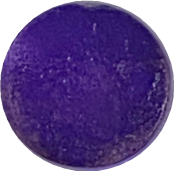

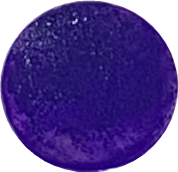

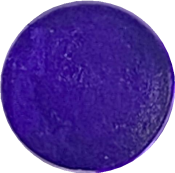

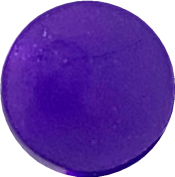

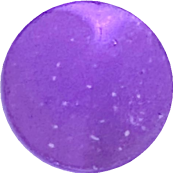


**Control Control+NIR**

**Uio-66**

**ICG@Uio-66 ICG@Uio-**

**66-UBI**

**ICG@Uio-66-+NIR**

**ICG@Uio-66-UBI+NIR**

**Fig. S4.** Inhibition effect of ICG@Uio-66-UBI NPs on *F. nucleatum, P. gingivalis and S. gordonii* biofilm formation. (a) Statistical data for biofilm biomass of *F. nucleatum*. (b) Crystal violet staining of *F.nucleatum* biofilms processed with different NPs. (c) Statistical data for biofilm biomass of *P. gingivalis*. (d) Crystal violet staining of *P. gingivalis* biofilms processed with different NPs. (e) Statistical data for biofilm biomass of *S. gordonii*. (f) Crystal violet staining of *S. gordonii* biofilms processed with different NPs. n=5, *p < 0.05, **p < 0.01, ***p < 0.001, ****p < 0.0001, ns, not significant.

**Control Control+NIR Uio-66 ICG@Uio-66**

**ICG@Uio-66-UBI**

**ICG@Uio-66+NIR**

**ICG@Uio-66-UBI+NIR**


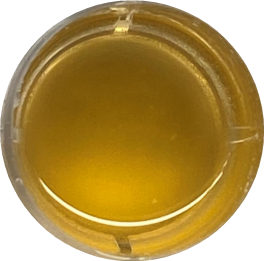

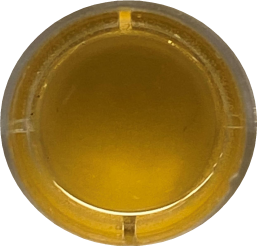

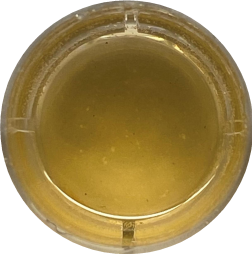

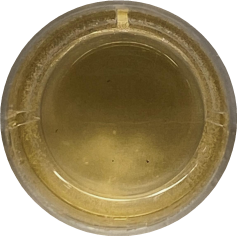

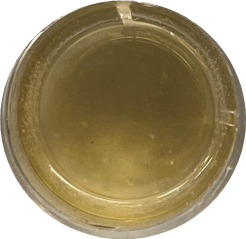


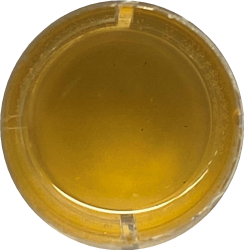

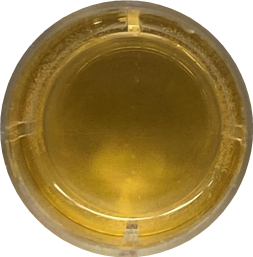


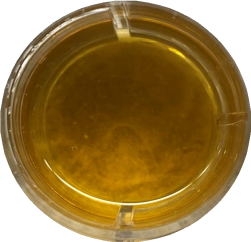

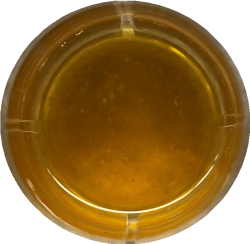

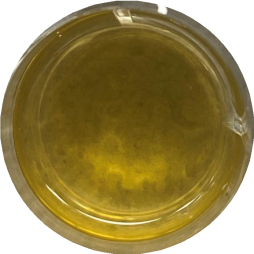

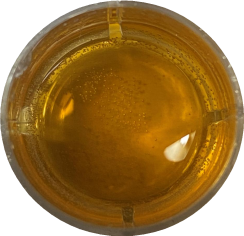


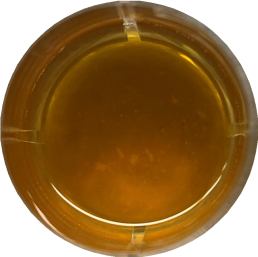

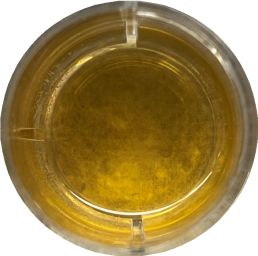

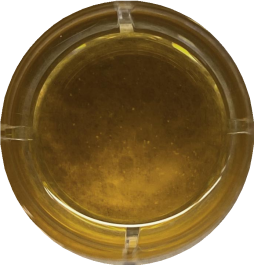

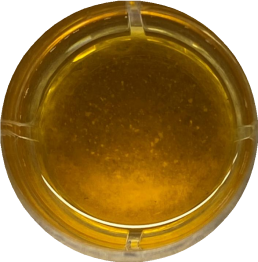

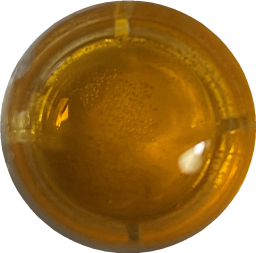

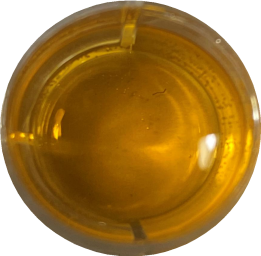

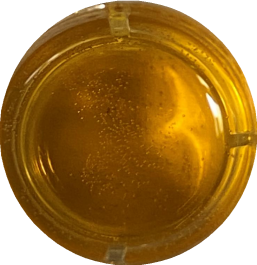

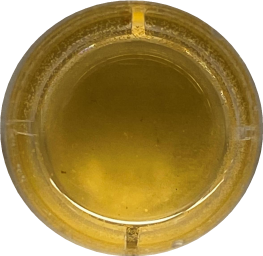

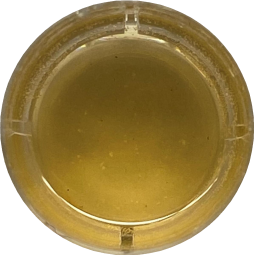

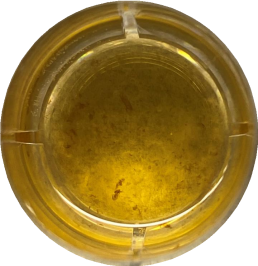


**Fig. S5**. EPS staining of *F. nucleatum P. gingivalis* and *S. gordonii*.

# a

**106**

# b

**106**


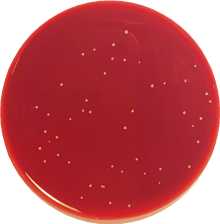

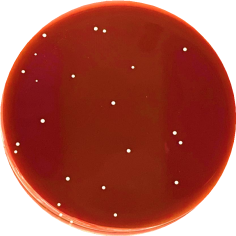

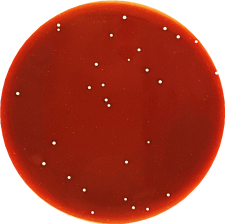

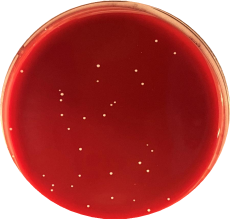

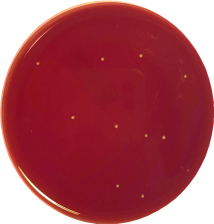

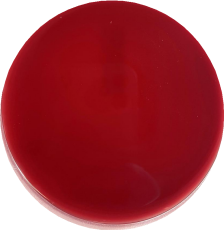

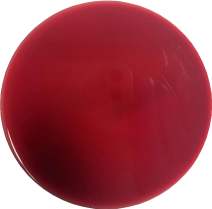

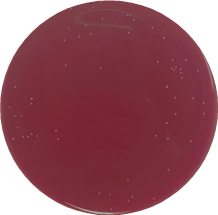

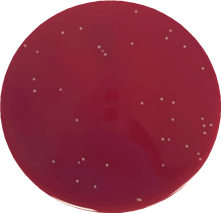

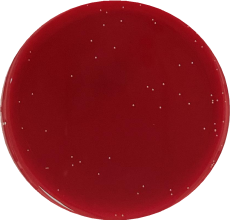

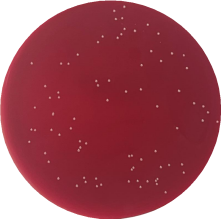

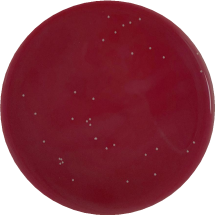

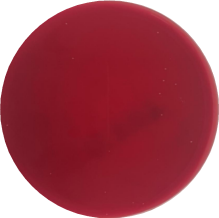

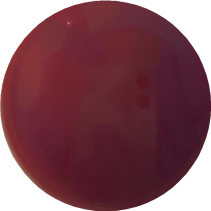


**Fig. S6**. Inhibition effect of ICG@Uio-66-UBI NPs on *F. nucleatum and P. gingivalis* biofilm formation. The images of *F. nucleatum* (a) and *P. gingivalis* (b) *clones*.

**a GO Enrichment b**

**KEGG Enrichment**


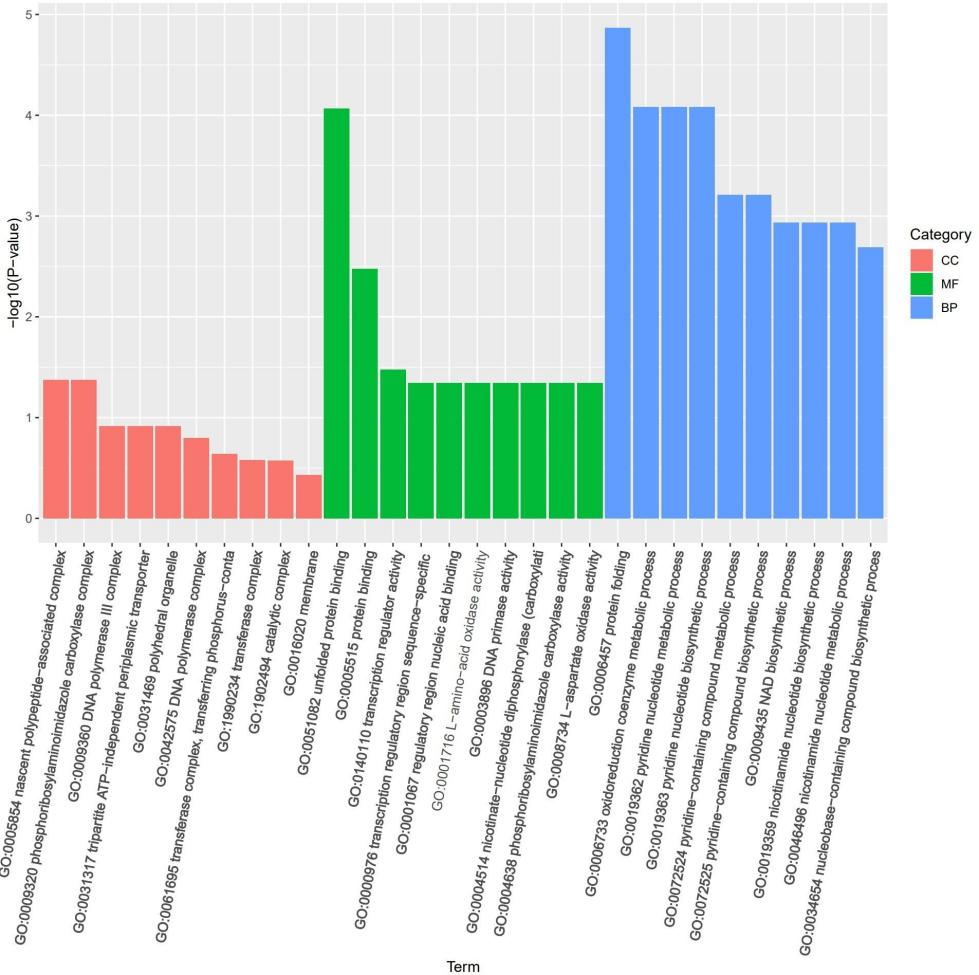


**Fig. S7.** RNA-seq analysis of *F. nucleatum* treated with ICG@Uio-66-UBI. (a) and (b) GO enrichment analysis of differentially expressed genes between the Control and ICG@Uio-66-UBI groups.

# a

**b C**

**Fig. S8.** Inhibition mechanisms of ICG@Uio-66-UBI NPs on *P. gingivalis* biofilm formation. (a) RT-qPCR analysis of the relative mRNA level of virulence factor genes in *P. gingivalis* biofilms. (b) Schematic representation of potential pathogenic mechanisms of virulence factors in *P. gingivalis*. (c) ICG@Uio-66-UBI inhibits secretion of AI-2 signaling molecule by *P. gingivalis*. n=5, *p <0.05, **p <0.01, ***p < 0.001, ****p < 0.0001, ns, not significant.

# a

**b**

**Fig. S9.** Inhibition mechanisms of ICG@Uio-66-UBI NPs on *S. gordonii* biofilm formation. (a) RT-qPCR analysis of the relative mRNA level of glycometabolic factor genes in *S. gordonii* biofilms. (b) ICG@Uio-66-UBI inhibits secretion of AI-2 signaling molecule *S. gordonii* . n=5, *p <0.05, **p <0.01, * **p < 0.001, ****p < 0.0001, ns, not significant.

# a b

**Fn**

**ICG@Uio-66-UBI**

**ICG@Uio-66-UBI+AI-2**

**ICG@Uio-66-UBI+AI-**

**ICG@Uio-66-UBI+ ICG@Uio-66-UBI+**

**2+20mM D-Gal AI-2+100mM D-Gal AI-2+200mM D-Gal**

**C**

**Fn**

**ICG@Uio-66-UBI+AI-2**

**+200mM D-Gal**

100μm

| **ICG@Uio-66-UBI** | **ICG@Uio-66-UBI+AI-2** | **ICG@Uio-66-UBI+AI-2**  **+20mM D-Gal** | **ICG@Uio-66-UBI+AI-2**  **+100mM D-Gal** |
| --- | --- | --- | --- |

**Fig. S10**. Inhibitory effect of D-galactose on *F. nucleatum* biofilm formation. (a) Statistical data for biofilm biomass of *F. nucleatum.* (b) Crystal violet staining of *F.nucleatum* biofilms processed in different groups. (c) The 3D live/dead images of *F.nucleatum* biofilms (dead bacteria, stained red; live bacteria, stained green)*.* n=5, *p <0.05, **p

<0.01, ***p < 0.001, ****p < 0.0001, ns, not significant.

# b

**l**

**Pg**

**ICG@Uio-66-UBI**

**ICG@Uio-66-UBI+ AI-2**

**ICG@Uio-66-UBI+ AI- ICG@Uio-66-UBI+**

**ICG@Uio-66-UBI+**

**2+20mM D-Gal AI-2+100mM D-Ga AI-2+200mM D-Gal**

**a**

**C**

**Fig. S11.** Inhibitory effect of D-galactose on *P. gingivalis* biofilm formation. (a) Statistical data for biofilm biomass of *P. gingivalis.* (b) Crystal violet staining of *P. gingivalis* biofilms processed in different groups. (c) The 3D live/dead images of *P. gingivalis* biofilms (dead bacteria, stained red; live bacteria, stained green)*.*n=5, *p <0.05, **p <0.01, ***p < 0.001,

**ICG@Uio-66-UBI**

**ICG@Uio-66-UBI+AI-2**

**ICG@Uio-66-UBI+AI-2**

**+20mM D-Gal**

**ICG@Uio-66-UBI+AI-2**

**+100mM D-Gal**

100μm

**ICG@Uio-66-UBI+AI-2**

**+200mM D-Gal**

**Pg**

****p < 0.0001, ns, not significant.

# a b

**l**

**Sg**

**ICG@Uio-66-UBI**

**ICG@Uio-66-UBI+ AI-2**

**ICG@Uio-66-UBI+ AI- ICG@Uio-66-UBI+**

**ICG@Uio-66-UBI+**

**2+20mM D-Gal AI-2+100mM D-Ga AI-2+200mM D-Gal**

**c**

| **Sg** | **ICG@Uio-66-UBI** | **ICG@Uio-66-UBI+AI-2** | **ICG@Uio-66-UBI+AI-2**  **+20mM D-Gal** | **ICG@Uio-66-UBI+AI-2**  **+100mM D-Gal** |
| --- | --- | --- | --- | --- |

**ICG@Uio-66-UBI+AI-2**

**+200mM D-Gal**

100μm

**Fig. S12.** Inhibitory effect of D-galactose on *S. gordonii* biofilm formation. (a) Statistical data for biofilm biomass of *S. gordonii.* (b) Crystal violet staining of *S. gordonii* biofilms processed in different groups. (c) The 3D live/dead images of *S. gordonii* biofilms (dead bacteria, stained red; live bacteria, stained green)*.*n=5, *p <0.05, **p <0.01, ***p < 0.001,

****p < 0.0001.

# a

**Control ICG @Uio-66 ICG @Uio-66-UBI ICG @Uio-66+NIR ICG@Uio-66-UBI+NIR**

**DAPI**

# b

**IL-6**

**DAPI**

**C**

**TNF-α**

**DAPI**

50μm

**Fig. S13.** Immunofluorescence staining of (a) IL-6, (b) TNF-α and (c) iNOS in a treatment model.

**iNOS**

**Fig. S14.** Corresponding analysis of positive cells expression of NF-κB/p65. n = 5 *p < 0.05, **p < 0.01,

***p < 0.001, ****p < 0.0001.

# a b

Control ICG@Uio-66-UBI+NIR

**Fig. S15.** TEM photomicrographs of *P. gingivalis* in (a) Control group and (b) ICG@Uio-66-UBI+NIR group (red arrow represent bacteria cell wall became ruptured).
